# The murine meninges acquire lymphoid tissue properties and harbour autoreactive B cells during chronic *Trypanosoma brucei* infection

**DOI:** 10.1371/journal.pbio.3002389

**Published:** 2023-11-20

**Authors:** Juan F. Quintana, Matthew C. Sinton, Praveena Chandrasegaran, Lalit Kumar Dubey, John Ogunsola, Moumen Al Samman, Michael Haley, Gail McConnell, Nono-Raymond Kuispond Swar, Dieudonné Mumba Ngoyi, David Bending, Luis de Lecea, Annette MacLeod, Neil A. Mabbott

**Affiliations:** 1 Lydia Becker Institute of Immunology and Inflammation, University of Manchester, United Kingdom; 2 Division of Immunology, Immunity to Infection and Health, Manchester Academic Health Science Centre, University of Manchester, United Kingdom; 3 School of Biodiversity, One Health, Veterinary Medicine (SBOHVM), College of Medical, Veterinary and Life Sciences, University of Glasgow, Glasgow United Kingdom; 4 Division of Cardiovascular Sciences, University of Manchester, United Kingdom; 5 Queen Mary University of London, London, United Kingdom; 6 Strathclyde Institute of Pharmacy and Biomedical Sciences (SIPBS), University of Strathclyde, Glasgow, United Kingdom; 7 Department of Parasitology, National Institute of Biomedical Research, Kinshasa, Democratic Republic of the Congo; 8 Institute of Immunology and Immunotherapy, College of Medical and Dental Sciences, University of Birmingham, Birmingham, United Kingdom; 9 Stanford University School of Medicine, Stanford, California, United States of America; 10 The Roslin Institute and Royal (Dick) School of Veterinary Studies, University of Edinburgh, Edinburgh, United Kingdom; Tokyo Medical and Dental University Medical Research Institute, JAPAN

## Abstract

The meningeal space is a critical brain structure providing immunosurveillance for the central nervous system (CNS), but the impact of infections on the meningeal immune landscape is far from being fully understood. The extracellular protozoan parasite *Trypanosoma brucei*, which causes human African trypanosomiasis (HAT) or sleeping sickness, accumulates in the meningeal spaces, ultimately inducing severe meningitis and resulting in death if left untreated. Thus, sleeping sickness represents an attractive model to study immunological dynamics in the meninges during infection. Here, by combining single-cell transcriptomics and mass cytometry by time-of-flight (CyTOF) with in vivo interventions, we found that chronic *T*. *brucei* infection triggers the development of ectopic lymphoid aggregates (ELAs) in the murine meninges. These infection-induced ELAs were defined by the presence of ER-TR7^+^ fibroblastic reticular cells, CD21/35^+^ follicular dendritic cells (FDCs), CXCR5^+^ PD1^+^ T follicular helper-like phenotype, GL7^+^ CD95^+^ GC-like B cells, and plasmablasts/plasma cells. Furthermore, the B cells found in the infected meninges produced high-affinity autoantibodies able to recognise mouse brain antigens, in a process dependent on LTβ signalling. A mid-throughput screening identified several host factors recognised by these autoantibodies, including myelin basic protein (MBP), coinciding with cortical demyelination and brain pathology. In humans, we identified the presence of autoreactive IgG antibodies in the cerebrospinal fluid (CSF) of second stage HAT patients that recognised human brain lysates and MBP, consistent with our findings in experimental infections. Lastly, we found that the pathological B cell responses we observed in the meninges required the presence of *T*. *brucei* in the CNS, as suramin treatment before the onset of the CNS stage prevented the accumulation of GL7^+^ CD95^+^ GC-like B cells and brain-specific autoantibody deposition. Taken together, our data provide evidence that the meningeal immune response during chronic *T*. *brucei* infection results in the acquisition of lymphoid tissue-like properties, broadening our understanding of meningeal immunity in the context of chronic infections. These findings have wider implications for understanding the mechanisms underlying the formation ELAs during chronic inflammation resulting in autoimmunity in mice and humans, as observed in other autoimmune neurodegenerative disorders, including neuropsychiatric lupus and multiple sclerosis.

## Abstract 2

This study shows that chronic brain infection with African trypanosomes induces broad meningeal responses resulting in the development of ectopic lymphoid aggregates containing autoreactive B cells; myelin basic protein is one of the autoantigens detected by autoreactive B cells, consistent with the cortical demyelination observed in experimental infections.

## Introduction

The meningeal space is rapidly being recognised as a critical site for immunological responses in the central nervous system (CNS) under homeostasis [1–3], aging [4], and as a consequence of insults such as traumatic brain injury [5] and infection [6–8]. The extracellular protozoan parasite *Trypanosoma brucei*, which causes human African trypanosomiasis (HAT; sleeping sickness in humans) and animal African trypanosomiasis (Nagana in domestic animals) accumulates in the CNS and meningeal spaces triggering severe meningitis [9,10]. This culminates in the development of a wide range of debilitating neurological disorders [9,11–13]. These symptoms are diverse and include fatigue, altered sleep and circadian patterns, tremors, motor weakness, epilepsy, paralysis of one or more extremities, and Parkinson-like abnormal body movements [14–16]. Consistent with clinical data from humans, experimental trypanosomiasis in mice also results in chronic infection, leading to altered behaviour [17–20]. Thus, murine infection with *T*. *brucei* is a useful model to investigate meningeal responses to infection.

Chronic inflammatory processes are known to result in the formation of ectopic lymphoid aggregates (ELAs) [21–24]. Indeed, ELAs have been reported in a wide range of autoimmune disorders, including those affecting the CNS such as neuropsychiatric lupus [25] and multiple sclerosis [26]. The diverse cytokine and chemokine repertoire found in chronically inflamed tissues, including lymphotoxin-α/-β (LTα and LTβ) and CXCL13, help to create interactive niches needed to generate such structures [21–24]. Stromal LTβ receptor (LTβR) signalling is important in generating the microarchitecture required for efficient antigen presentation and follicle organisation, which typically includes collagen-rich reticular cords that serve as channels for cellular trafficking, immunological synapses, and B cell affinity maturation [27]. Similarly, CXCL13 is an important chemokine for defining local gradients controlling B cell domains, typically in proximity to follicular dendritic cells (FDCs) and CD4^+^ T follicular helper cells (T_FH_) inducing the formation of germinal centres (GC), in which B cells undergo affinity maturation and somatic hypermutation to generate high-affinity antibodies [21,23,25]. These reactions are typically restricted to secondary lymphoid organs such as the spleen and lymph nodes, but can occur ectopically in response to chronic inflammation, and may result in pathological consequences such as the formation of autoreactive antibodies, as recently described for multiple sclerosis and neuropsychiatric lupus [23,25,26,28].

In secondary lymphoid organs, including the spleen and lymph nodes, lymphatic vessels act as conduits for the transport of tissue-derived antigens and dendritic cells (DCs) to lymph nodes, where naïve and memory T cells are optimally positioned for the detection of their cognate antigen [29–31]. Similarly, immune complexes can be acquired by macrophages in the subcapular space in lymph nodes and transferred directly to FDCs and B cells [32]. However, several key findings in recent years have led to a better understanding of the role of lymphatic vessels in the dura mater layer of the meninges. For example, the meningeal lymphatic vessels can convey macromolecular complexes and immune cells from the meninges and cerebrospinal fluid (CSF) to the deep cervical lymph nodes [4,33,34]. However, it is also plausible that extramedullary reactions may take place locally in the meningeal spaces and brain borders, as reported recently in neuropsychiatric lupus [25] and multiple sclerosism [28]. Whether the same extramedullar immunological reactions in the brain and/or meninges, reminiscent of those taking place in secondary lymphoid tissues, can be triggered by chronic, unresolved infections is uncertain.

Here, we investigated how the meningeal transcriptional environment is altered during *T*. *brucei* infection, using a combination of single-cell transcriptomics and mass cytometry by time-of-flight (CyTOF). We found that chronic *T*. *brucei* infection in the meningeal space results in a broad rearrangement of the immune landscape in the murine meninges, with a significant increase in the frequency of innate (mononuclear phagocytes (MNPs) and granulocytes) and adaptive (T, NKT, and B cells) immune cells. Furthermore, we identified a population of autoreactive B cells in the meningeal spaces, including the leptomeninges. These autoreactive B cells were able to recognise mouse brain antigens and deposit high-affinity IgG antibodies in several brain areas including the hippocampus and cortex, and this deposition was associated with cortical and white mater demyelination. We also detected significant levels of autoreactive IgM and IgG antibodies in the CSF of HAT patients with inflammatory encephalopathy. Furthermore, using a targeted screening approach we identified myelin basic protein (MBP) as one of the host antigens detected by autoreactive IgG antibodies in mouse serum and human CSF collected during the chronic stage of the infection. Taken together, this study demonstrates that the meningeal landscape acquires lymphoid tissue-like properties resulting in the formation of autoreactive B cells. These results imply that the chronic brain inflammation induced by African trypanosomes results in an autoimmune disorder affecting the brain as observed in other neurological disorders of unknown aetiology such as neuropsychiatric lupus and multiple sclerosis. We anticipate that the data presented here will pave the way to understanding how chronic meningitis results in impaired peripheral tolerance and the development of autoimmunity in the context of chronic infections.

## Materials and methods

### Ethical statement

All animal experiments were approved by the University of Glasgow Ethical Review Committee and performed in accordance with the Home Office guidelines, UK Animals (Scientific Procedures) Act, 1986 and EU directive 2010/63/EU. All experiments were conducted under SAPO regulations and UK Home Office project licence number PP4863348 to Annette Macleod. The in vivo work presented in this study was conducted at 30 days postinfection (dpi) and correlated with increased clinical scores and procedural severity. The archived human CSF samples from gambiense HAT patients from North Uganda used in this study were collected by Professor Wendy Bailey (Liverpool School of Tropical Medicine, United Kingdom). Ethical approval was given to Prof. Bailey by the Liverpool School of Tropical Medicine, UK, for sample collection and patients were provided with a written consent. We received ethical approved by the University of Glasgow MVLS Ethics Committee for Non-Clinical Research Involving Human Subjects (Reference no. 200120043) for the use of human archived samples. The CSF from healthy donors was obtained from the University of Edinburgh Brain and Tissue Bank and received ethical approval from the University of Edinburgh (REC 21/ES/0087).

### Murine infections with *Trypanosoma brucei*

Six- to 8-week-old female C57BL/6J mice (JAX, stock 000664) and C57BL/6-Tg(Nr4a1-EGFP/Cre)820Khog/J strain, also known as Nur77^GFP^ reporter mice (JAX, stock 016617), or the Nur77^Tempo^ reporter mouse line (kindly provided by Dr. David Bending), were inoculated by intraperitoneal injection with approximately 2 × 10^3^ parasites of strain *T*. *brucei brucei* Antat 1.1E [35]. Parasitaemia was monitored by regular sampling from tail venesection and examined using phase microscopy and the rapid “matching” method [36]. Uninfected mice of the same strain, sex, and age served as uninfected controls. Mice were fed ad libitum and kept on a 12 h light–dark cycle. All the experiments were conducted between 8 h and 12 h. When using the *Nur77*^GFP^ or the *Nur77*^Tempo^ reporter mice, sample acquisition and analysis was conducted without ex vivo stimulation to preserve the TCR-dependent fluorescent reporter signal found in the tissue. For sample collection, we focussed on 30 dpi, as this has previously been shown to correlate with parasite infiltration in the epidural space [10,11]. Culture-adapted *T*. *brucei* Antat 1.1E whole cell lysates were prepared as followed. Parasites were cultured in HMI-9 supplemented with 10% FBS and 1% Penicillin/Streptomycin were grown at 37°C and 5% CO_2_ and harvested during the log phase. The parasites were harvested by centrifugation (800 g for 10 min at 4°C), washed 3 times in 1× PBS (Gibco) supplemented with cOmplete protease Inhibitor cocktail (Roche), and sonicated with 5 pulses of 10 s each. The resulting lysate was cleared by centrifugation (3,000g for 10 min at 4°C to remove cell debris), and the protein concentration of the cleared supernatant was measured using the Qubit protein kit (Thermo) and kept at −80°C until usage for ELISPOT and ELISA. For LTBR-Ig treatment, mice were inoculated with 1 μg/μl of either LTBR-Ig or IgG2a i.p. (100 μl/mouse) for 4 consecutive days prior to infection, and then every 7 dpi until culling. Preparation of single-cell suspension from skull meninges for single-cell RNA sequencing.

### Tissue processing and preparation of single-cell suspension

Single-cell dissociations for scRNAseq experiments were performed as follow. Animals were infected for 30 days (*n* = 2 mice/pool, 2 independent pools per experimental condition), after which skullcap meninges were harvested for preparation of single-cell suspensions. Uninfected animals were also included as naive controls (*n* = 3 mice/pool, 2 pools analysed). Briefly, all mice were killed by rapid decapitation following isoflurane anaesthesia, within the same time (between 7:00 and 9:00 AM). To discriminate circulating versus brain-resident immune cells, we performed intravascular staining of peripheral CD45^+^ immune cells, as previously reported [37]. Briefly, a total of 2 μg of anti-CD45-PE antibody (in 100 μl of 1× PBS) was injected intravenously 3 min prior culling. Mice were euthanised as described above and transcardially perfused with ice-cold 0.025% (wt/vol) EDTA in 1× PBS. The excised meninges were enzymatically digested with Collagenase P (1 mg/ml) and DNAse I (1 mg/ml; Sigma) in 1× PBS (HSBB) (Invitrogen) for approximately 30 min at 37°C. Single-cell suspensions were passed through 70 μm nylon mesh filters to remove any cell aggregates, and the circulating CD45-PE^+^ cells were removed from the single-cell suspension using magnetic sorting with anti-PE microbeads (Miltenyi Biotec) according to manufacturer’s recommendations (**S1A Fig**).

### Mass cytometry sample processing

Single-cell suspension from meninges were prepared as described above and resuspended in Dubecco’s Modified Eagle Medium (DMEM) to a concentration of 1 × 10^6^ cells/ml. Cells were activated for 6 h in a round-bottom 96-well plate using Cell Activation Cocktail (containing with Brefeldin A) (BioLegend, San Diego, United States of America) as per the manufacturer’s recommendations. Plates were then centrifuged at 300 × g for 5 min and the pellets resuspended in 50 μl of Cell-ID Cisplatin-195Pt viability reagent (Standard BioTools, San Francisco, USA), and incubated at room temperature for 2 min. Cells were washed twice in Maxpar Cell Staining Buffer (Standard BioTools, San Francisco, USA) and centrifuged at 300 × g at room temperature for 5 min. The CD16/CD32 receptors were then blocked by incubating with a 1/50 dilution of TruStain FcX (BioLegend, San Diego, USA) in PBS at room temperature for 15 min. An antibody cocktail was prepared from the Maxpar Mouse Sp/LN Phenotyping Panel Kit (Standard BioTools, San Francisco, USA), with and additional antibody against IgM. Cells were incubated with antibodies for 60 min, on ice before washing 3 times in Maxpar Cell Staining Buffer (Standard BioTools, San Francisco, USA) as previously. Following staining, cells were fixed in 2% paraformaldehyde (PFA) overnight at 4°C. Cells were then washed twice with 1× eBioscience Permeabilization Buffer (Invitrogen, Waltham, USA) at 800 × g at room temperature for 5 min. The pellets were resuspended in intracellular antibody cocktail and incubated at room temperature for 45 min. Cells were washed 3 times in Maxpar Cell Staining Buffer (Standard BioTools, San Francisco, USA) at 800 × g. The cells were then resuspended in 4% PFA at room temperature for 15 min, before collecting the cells at 800 × g and resuspending in Cell-ID Intercalator-Ir (Standard BioTools, San Francisco, USA). Finally, the cells were barcoded by transferring the stained cells to a fresh tube containing 2 μl of palladium barcode from the Cell-ID 20-Plex Pd Barcoding Kit (Standard BioTools, San Francisco, USA). Cells were then frozen in a freezing solution (90% FBS and 10% DMSO) before shipping to the Flow Cytometry Core Facility at the University of Manchester for data acquisition. Sample analysis was conducted using Cytobank and custom-built, python-based analysis scripts developed in house (**S1B and S1C Fig for QC results**). The antibodies used for labelling were as follow (Standard Biotools, cat No. 201306): Ly6G/C [Gr1] (141^Pr^, clone RB6-8C5, 1/100), CD11c (142^Nd^, clone N418, 1/100), CD69 (145^Nd^, clone H1.2F3, 1/100), CD45 (147^Sm^, clone 30-F11, 1/200), CD11b (148^Nd^, clone M1/70, 1/100), CD19 (149^Sm^, clone 6D5, 1/100), CD3e (152^Sm^, clone 145-2C11, 1/100), TCRβ (169^Tm^, clone H57-597, 1/100), CD44 (171^Yb^, clone IM7, 1/100), CD4 (172^Yb^, clone RM4-5, 1/100), IgM (151^Eu^, clone RMM-1, 1/100), IFNγ (165^Ho^, clone XMG1.2, 1/100).

### Single-cell transcriptomics analysis of murine meninges

The single-cell suspension obtained from murine meninges after the CD45-PE depletion step was diluted to approximately 1,000 cells/μl (in 1× phosphate buffered saline supplemented with 0.04% BSA) and kept on ice until single-cell capture using the 10X Chromium platform. The single-cell suspensions were loaded onto independent single channels of a Chromium Controller (10X Genomics) single-cell platform. Briefly, approximately 25,000 single cells were loaded for capture using 10X Chromium NextGEM Single cell 3 Reagent kit v3.1 (10X Genomics). Following capture and lysis, complementary DNA was synthesised and amplified (12 cycles) as per the manufacturer’s protocol (10X Genomics). The final library preparation was carried out as recommended by the manufacturer with a total of 14 cycles of amplification. The amplified cDNA was used as input to construct an Illumina sequencing library and sequenced on a Novaseq 6000 sequencers by Glasgow polyomics.

### Read mapping, data processing, and integration

For FASTQ generation and alignments, Illumina basecall files (*.bcl) were converted to FASTQs using bcl2fastq. Gene counts were generated using Cell Ranger v.6.0.0 pipeline against a combined *Mus musculus* (mm10) and *Trypanosoma brucei* (TREU927) transcriptome reference. After alignment, reads were grouped based on barcode sequences and demultiplexed using the unique molecular identifiers (UMIs). The mouse-specific digital expression matrices (DEMs) from all 6 samples were processed using the R (v4.1.0) package Seurat v4.1.0 [38]. Additional packages used for scRNAseq analysis included dplyr v1.0.7, RColorBrewer v1.1.2 (http://colorbrewer.org), ggplot v3.3.5, and sctransform v0.3.3 [39]. We initially captured 20,621 cells mapping specifically against the *M*. *musculus* genome across all conditions and biological replicates, with an average of 30,407 reads/cell and a median of approximately 841 genes/cell (**S1 Table and S1D Fig**). The number of UMIs was then counted for each gene in each cell to generate the DEM. Low-quality cells were identified according to the following criteria and filtered out: (i) nFeature <200 or >4,000 genes; (ii) nCounts <200 or >4,000 reads; (iii) >20% reads mapping to mitochondrial genes; (iv) >40% reads mapping to ribosomal genes; and (v) genes detected <3 cells. After applying this cutoff, we obtained a total of 19,690 high-quality mouse-specific cells with an average of 950 genes/cell (**S1 Table and S1D Fig**). High-quality cells were then normalised using the *SCTransform* function, regressing out for total UMI and genes counts, cell cycle genes, and highly variable genes identified by both Seurat and Scater packages, followed by data integration using *IntegrateData* and *FindIntegrationAnchors*. For this, the number of principal components were chosen using the elbow point in a plot ranking principal components and the percentage of variance explained (30 dimensions) using a total of 5,000 genes and SCT as normalisation method.

### Cluster analysis, marker gene identification, subclustering, and cell–cell interaction analyses

The integrated dataset was then analysed using *RunUMAP* (10 dimensions), followed by *FindNeighbors* (10 dimensions, reduction = “pca”) and *FindClusters* (resolution = 0.7). The resolution was chosen based on in silico analysis using *Clustree* [40] (**S1E Fig**). With this approach, we identified a total of 19 cell clusters. The cluster markers were then found using the *FindAllMarkers* function (logfc.threshold = 0.25, assay = “RNA”). To identify cell identity confidently, we employed a supervised approach. This required the manual inspection of the marker gene list followed by and assignment of cell identity based on the expression of putative marker genes expressed in the unidentified clusters. This was particularly relevant for immune cells detected in our dataset that were not found in the reference atlases used for mapping. A cluster name denoted by a single marker gene indicates that the chosen candidate gene is selectively and robustly expressed by a single-cell cluster and is sufficient to define that cluster (e.g., *Cd79a*, *Cd4*, *C1qa*, *Cldn5*, among others). When manually inspecting the gene markers for the final cell types identified in our dataset, we noted the co-occurrence of genes that could discriminate 2 or more cell types (e.g., DCs, MNPs, fibroblasts). To increase the resolution of our clusters to help resolve potential mixed cell populations embedded within a single cluster and, we subset fibroblasts, DCs, and MNPs and analysed them individually using the same functions described above. In all cases, upon subsetting, the resulting objects were reprocessed using the functions *FindVariableFeatures*, *RunUMAP*, *FindNeighbors*, and *FindClusters* with default parameters. The number of dimensions used in each case varied depending on the cell type being analysed but ranged between 5 and 10 dimensions. Cell type-level differential expression analysis between experimental conditions was conducted using the *FindMarkers* function (*min*.*pct* = 0.25, *test*.*use* = Wilcox) and (*DefaultAssay* = “SCT”). For cell–cell interaction analyses, we used CellPhoneDB [41] and NicheNet [42] with default parameters using “mouse” as a reference organism, comparing differentially expressed genes between experimental conditions (*condition_oi* = “Infected”, *condition_reference* = “Uninfected”). Pathways analysis for mouse genes was conducted using STRING ^26^ with default parameters. Module scoring were calculated using the *AddModuleScore* function to assign scores to groups of genes of interest (*Ctrl* = 100, *seed* = NULL, *pool* = NULL), and the scores were then represented in feature plots. This tool measures the average expression levels of a set of genes, subtracted by the average expression of randomly selected control genes. The complete gene list used for module scoring derived from previous publications [25] or from the MatrisomeDB [43]. Once defined, the collated gene list was used to build the module scoring.

### Whole mount meningeal preparation and immunofluorescence

After euthanasia, the skull caps were carefully removed using fine tweezers and scissors and placed immediately in 10% neutral buffered formalin (NFB) for 10 min at room temperature. Coronal brain sections were also fixed as above, embedded in paraffin, and processed for Luxol fast blue (LFB) used as a proxy to measure the levels of myelin. Following fixation of the skull caps, for immunofluorescence staining, the meninges were detached from the skull caps using a stereotactic microscope and kept at 4°C in 1× PBS containing 0.025% sodium azide until imaging (no longer than 1 week). For histological analysis, the dura meninges were left attached to the skull and the samples were decalcified prior to embedding in paraffin using neutral EDTA; 2 to 3 μm skull sections were then prepared for in situ hybridisation experiments or for Masson’s trichrome staining. For immunofluorescence staining, sections were blocked with blocking buffer (1× PBS supplemented with 5% foetal calf serum and 0.2% Tween 20) and incubated with the following primary antibodies at 4°C overnight: REAfinity anti-mouse FITC CD21/35 (Miltenyi, 1:50), rat anti-mouse ER-TR7 (Novus Biologicals, 1:100), REAfinity anti-mouse PE CD3 (Miltenyi, 1:100), REAfinity anti-mouse APC B220 (Miltenyi, 1:100), anti-mouse CD138 PE (BD Bioscience, 1:100). For the detection of ER-TR7, we used an anti-rat antibody coupled with PE (Thermo, 1:500) for 1 h at room temperature. All the antibodies were diluted in blocking buffer. Slides were mounted with Vectashield mounting medium containing DAPI for nuclear labelling (Vector Laboratories) and were visualised using an Axio Imager 2 (Zeiss). Single-molecule fluorescent in situ hybridisation (smFISH) experiments were conducted as follow. Briefly, to prepare tissue sections for smFISH, infected animals and naïve controls were anesthetized with isoflurane, decapitated and the skull caps containing the dura mater layer of the meninges were dissected and place on ice-cold 1× HBSS. The skulls were then fixed with 4% paraformaldehyde (PFA) at 4°C for 15 min, and then dehydrated in 50%, 70%, and 100% ethanol. After fixation, the skulls caps were decalcified, cut coronally, and embedded in paraffin; 5 μm skull cap sections were RNAscope 2.5 Assay (Advanced Cell Diagnostics) was used for all smFISH experiments according to the manufacturer’s protocols. We used RNAscope probes against mouse *Rarres2* on channel 1 (Cat No. 572581), *Cxcl13* on channel 2 (Cat. No. 406311-C2), and Ly6a on channel 3 (Cat. No 427571-C3). All RNAscope smFISH probes were designed and validated by Advanced Cell Diagnostics. For image acquisition, 16-bit laser scanning confocal images were acquired with a 63×/1.4 plan-apochromat objective using an LSM 710 confocal microscope fitted with a 32-channel spectral detector (Carl Zeiss). Lasers of 405 nm, 488 nm, and 633 nm excited all fluorophores simultaneously with corresponding beam splitters of 405 nm and 488/561/633 nm in the light path, and 9.7 nm binned images with a pixel size of 0.07 μm × 0.07 μm were captured using the 32-channel spectral array in Lambda mode. Single fluorophore reference images were acquired for each fluorophore and the reference spectra were employed to unmix the multiplex images using the Zeiss online fingerprinting mode. All fluorescent images were acquired with minor contrast adjustments where needed, and converted to grayscale, to maintain image consistency.

### Flow cytometry analysis and ex vivo stimulation of meningeal-dwelling T cells

To discriminate circulating versus brain-resident immune cells, we performed intravascular staining of peripheral CD45^+^ immune cells, as previously reported [37]. Briefly, a total of 2 μg of anti-CD45-APC-Cy7 antibody (clone 30-F11, in 100 μl of 1× PBS) was injected intravenously approximately 3 min prior culling. Mice were euthanised as described above and transcardially perfused with ice-cold 0.025% (wt/vol) EDTA in 1× PBS. Whole meninges were enzymatically digested with Collagenase P (1 mg/ml) and DNAse I (1 mg/ml; Sigma) in 1× PBS (HSBB) (Invitrogen) for approximately 30 min at 37°C, according to previously published protocols [44]. Single-cell suspensions were passed through 70 μm nylon mesh filters to remove any cell aggregates. The cell suspension was cleaned up and separated from myelin debris using a Percoll gradient. The resulting fraction was then gently harvested and used as input for ex vivo T cell stimulation or used as input for downstream flow cytometry analysis. Briefly, the resulting cell fraction was diluted to a final density of approximately 1 × 10^6^ cells/ml and seeded on a 96-well plate and stimulated with 1× cell Stimulation cocktail containing phorbol 12-myristate 13-acetate (PMA), Ionomycin, and Brefeldin A (eBioSciences) for 5 h at 37°C and 5% CO_2_. Upon stimulation, the cells were analysed for the expression of IL-21 and PD-1.

For flow cytometry analysis, meningeal single-cell suspensions were resuspended in ice-cold FACS buffer (2 mM EDTA, 5  U/ml DNAse I, 25 mM HEPES, and 2.5% foetal calf serum (FCS) in 1× PBS) and stained for extracellular markers. The list of flow cytometry antibodies used in this study was obtained from Biolegend and is presented in the table below. Samples were run on a flow cytometer LSRFortessa (BD Biosciences) and analysed using FlowJo software version 10 (Treestar). For intracellular staining, single-cell isolates from brain were stimulated as above in Iscove’s modified Dulbecco’s media (supplemented with 1× non-essential amino acids, 50 U/ml penicillin, 50 μg/ml streptomycin, 50 μm β-mercaptoethanol, 1 mM sodium pyruvate, and 10% FBS, Gibco). Cells were then permeabilized with a Foxp3/Transcription Factor Staining Buffer Set (eBioscience) and stained for 30 min at 4°C. The anti-mouse GP38 (1:100) and the LTβ (monoclonal antibody BBF6 [45]; 10 μg/ml) antibodies were kindly provided by Dr. Lalit Kumar Dubey (QMUL). For the detection of LTβ in CD4^+^ T cells, we used a goat anti-hamster (Armenian) IgG coupled to FITC as secondary antibody (Biolegend; 1:200). For the detection of GP38, we used a Syrian hamster-anti mouse GP38 followed by anti-Syrian hamster secondary antibody coupled to APC/alexa647 (Jackson ImmunoResearch; 1:100). We used the following commercially available antibodies from Biolegend: CD45-APC-Cy7 (clone 30-F11, 2 μg/100 μl 1× PBS i.v.), TER-119-APC-Cy7 (clone TER-119; 1/400), CD19-APC-Cy7 (clone 1D3/CD19; 1/400), F4/80-APC-Cy7 (clone BM8; 1/400), F4/80-PE Dazzle 594 (clone BM8; 1/400), CD3-APC (clone 17A2; 1/400), CD4-FITC (clone GK1.5; 1/400), PD1-BV711 (clone 29F.1A12; 1/400), CXCR5-BV421 (clone L138D7; 1/200), IL-21-PE (clone 3A3-N2; 1/200), CD45-BV711 (clone 30-F11; 1/400), CD31-BV421 (clone 8.1.1; 1/100), CD21/35-PE Dazzle 594 (clone 7E9; 1/100), MAdCAM-1-Alexa Fluor 488 (clone MECA-367; 1/100), CD19- Alexa Fluor 488 (clone 6D5; 1/400), CD138-PE (clone 281–2; 1/200), IgG-BV421 (clone Poly4053; 1/200), IgM-BV711 (clone RMM-1; 1/200), CD8a-BV421 (clone QA17A07; 1/400), I-A/I-E-PerCP-Cy5.5 (clone M5/114.15.2; 1/400).

### ELISPOT assays

ELISPOT tests to measure ex vivo the frequency of meningeal antibody secreting cells (ASCs) was performed using the ELISpot Flex IgM- and IgG-HRP (Mabtech) as followed. After generating single-cell suspensions from meningeal preparations, a total of 50,000 cells per well were seeded on 96-wells multiscreen-HA filter plates (Millipore) coated with 50 μg/ml of either whole *T*. *brucei*, prepared in house, or mouse brain lysate (Novus Biologicals) to determine the presence of *T*. *brucei*- and mouse brain-reactive ASCs, respectively. Wells coated with 50 μg/ml BSA (Sigma) were also included as negative controls. After seeding the cells, the plates were incubated for 16 h at 37°C and 5% CO_2_ covered in foil to avoid evaporation. In parallel, plates coated with 15 μg/ml affinity-purified goat anti-mouse IgM and IgG were also analysed in parallel to measure the frequency of total IgM and IgG ASCs. For this, we used a total of 25,000 cells per well and incubated as before. Spots were enumerated with an Immunospot analyser (CTL, Germany).

### Detection of autoreactive IgM and IgG by ELISA

Serum samples from naïve and infected animals at 30 dpi were used to examine the presence of mouse brain lysate-specific IgM and IgG using a colorimetric approach. For this purpose, polysorb ELISA plates (Biolegend) were coated overnight with 50 μg/ml either *T*. *brucei* Antat 1.1E whole cell lysate prepared in house, or mouse brain lysate (Novus Biologicals) in 1× coating buffer (Biolegend). After extensive washes with 1× ELISA washing buffer (Biolegend), total mouse IgM or IgG were detected in mouse serum (1:50 to 1:10,000 dilution in 1× PBS) or human CSF (1:400 in 1× PBS) by using Horseradish peroxidase-conjugated antibodies specific for mouse IgM (Thermo) or IgG (all isotypes; Sigma) using the recommended concentrations, and the resulting absorbance was detected at 450 nm using an ELISA Multiskan plate reader (Thermo).

### Detection of host antigens detected by autoantigens

Blood samples were collected by cardiac puncture from naïve mice (*n* = 3 mice) or at 30 dpi (*n* = 3 mice) were and place on EDTA tubes, from which serum was obtained. In parallel, we screened CSF samples collected from first stage sleeping sickness patients (*n* = 3 patients) and second stage patients (*n* = 4 patients). Due to ethical constraints, we did not have access to CSF samples from African healthy donors. Therefore, we obtained CSF samples from healthy Caucasian donors (*n* = 2 donors) from the University of Edinburgh Brain and Tissue Bank. Autoantibodies were assessed using a commercial microarray-based platform (GeneCopoeia). Briefly, mouse serum or human CSF samples were hybridised to distinct microarray spots containing 120 native host and viral antigens spotted onto nitrocellulose fibres (adhered to glass slides). Next, the slides were incubated with fluorescently coupled anti-IgG or anti-IgM secondary antibodies, and microarrays were scanned using a GenePix 4400A microarray scanner. Raw fluorescence data was normalised to PBS controls on each slide. The data presented in the heatmaps are normalised signal-to-noise ratios.

### Statistical analysis

All statistical analyses were performed using Graph Prism Version 8.0 for Windows or macOS, GraphPad Software (La Jolla, California, USA). The data distribution was determined by normality testing using the Shapiro–Wilk test. Where indicated, data were analysed by unpaired Student’s *t* test, Mann–Whitney test, or one-way analysis of variance (ANOVA). Data were considered to be significant where *p* < 0.05. For the in vivo experiments, we matched sex and age of the mice in experimental batches using a block design including randomisation of experimental units. Data collection and analysis were not performed blindly to the conditions of the experiment due to the specific requirements of the UK Home Office project licence.

## Results

### The murine meninges are colonised by a diversity of immune cells during chronic *T*. *brucei* infection

**Fig 1:**
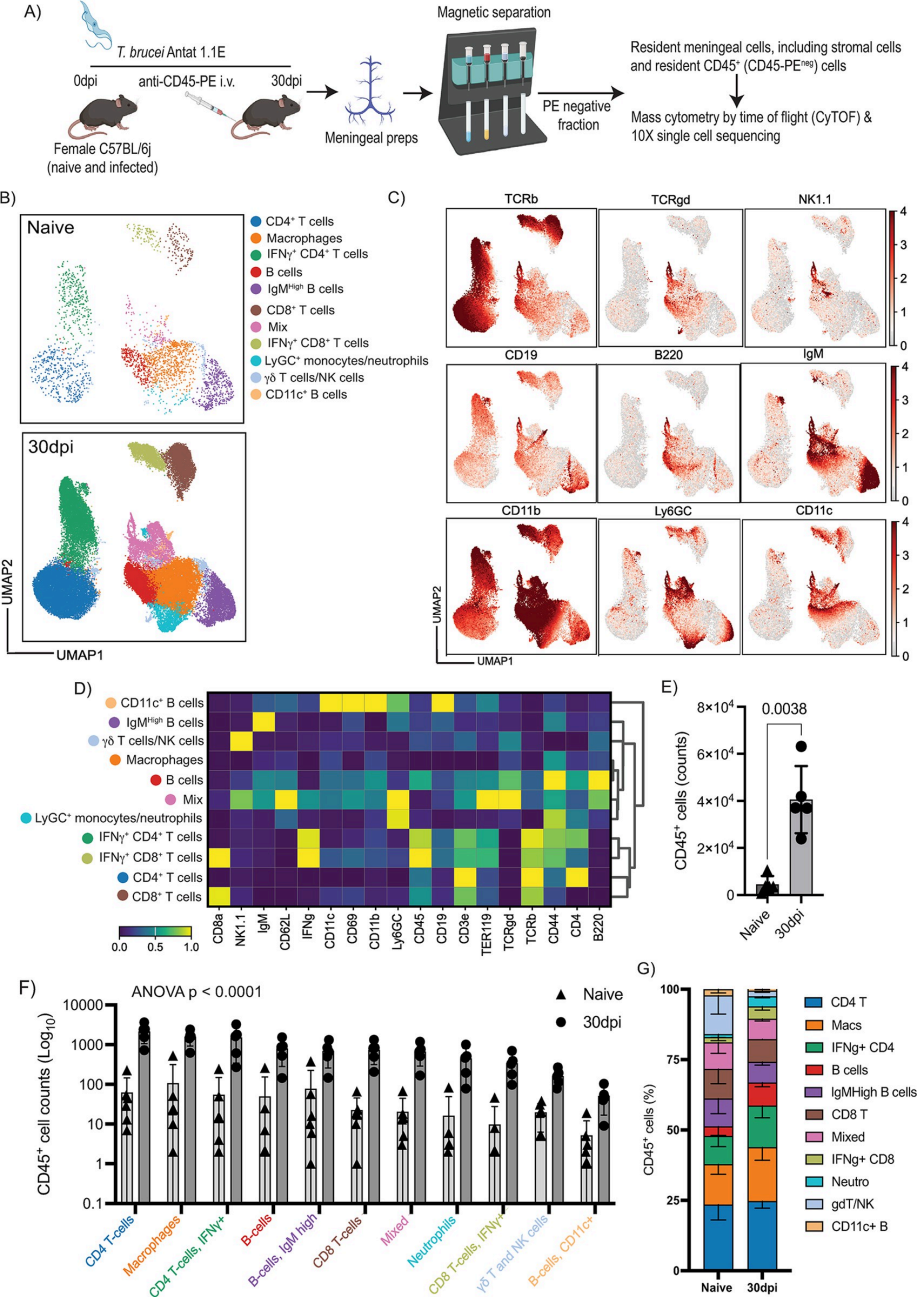
CyTOF confirms the expansion of innate and adaptive immune cells in the murine meninges during chronic *T*. *brucei* infection. **(A)** Overview of the experimental approach applied in this work. An anti-CD45-PE antibody was injected *i*.*v*. prior to cull, followed by magnetic sorting using anti-PE antibodies to obtain a fraction of resident meningeal cells that were used as input for single-cell transcriptomics using the 10X Chromium platform. Images generated with BioRender. **(B)** UMAP visualisation of the CyTOF immunophenotyping in murine meninges from naïve and animals at 30 dpi. The UMAP plot represents pooled data from *n* = 5–6 mice/group. **(C)** Normalised protein expression level of markers used to define T cells and NK cells (TCRβ, TCRγδ, NK1.1), B cells (CD19, B220, IgM), and myeloid cells (CD11b, Ly6G/C, and CD11c). **(E)** Quantification of CD45^+^ cells in the murine meninges by CyTOF at 30 dpi (*n* = 5 mice/group). Parametric two-sided *T* student test: a *p* value < 0.05 was considered significant. Supporting data in S1 Data file. **(D)** Unsupervised cell annotation from CyTOF data using a combination of several marker genes. The expression level is normalised to the average of the expression within the group. **(F)** Quantification of the different populations of immune cells in the naïve and infected murine meninges (*n* = 5–6 mice/group). A parametric ANOVA test with multiple comparison was used to estimate statistically significant pairwise comparisons. A *p* value < 0.05 is considered significant. Data in all panels are expressed as mean ± SD. Data points indicate biological replicates for each panel. Supporting data in S2 Data file. **(G)** Bar chart depicting the frequency of the different immune populations identified in the murine meninges by CyTOF. Supporting data in S3 Data file. CyTOF, cytometry by time of flight; dpi, days postinfection; PE, phycoerythrin; UMAP, uniform manifold approximation and projection.

We and others have shown an increase in meningeal infiltration and meningitis during the chronic stage (25 dpi onwards) in experimental infections with *T*. *brucei* [10,13]. Previous studies have shown that mouse meninges are colonised by CD2^+^ T cells and CD11c^+^ DCs during chronic *T*. *brucei* infection [9], although a catalogue of the immune interactions spanning beyond these compartments is lacking. To fill this gap in knowledge, we used an integrative multi-omics approach that combined CyTOF and 10X Chromium single-cell transcriptomics (**Fig 1A**) to understand the complexity of the immune interactions taking place in the meningeal space during chronic *T*. *brucei* infection. In addition to unbiasedly cataloguing the cells involved in this process, this approach also allowed us to identify transcriptional pathways involved in the antiparasitic responses in the meninges with as much resolution as possible. We focussed on characterising the dura mater as this has been previously shown to contain the vast majority of the meningeal CD45^+^ immune cells [42], as well as parasites during the chronic stages of the infection [9]. To ensure we captured the diversity of resident immune cells and meningeal stroma with as much confidence as possible, we selectively removed all circulating CD45^+^ immune cells using a magnetic sorting approach (**Figs 1A and S1A**). In brief, we labelled circulating CD45^+^ cells by intravenous injection with an anti-CD45^+^ antibody coupled to phycoerythrin (PE). All the circulating CD45^+^ were then isolated using an anti-PE antibody coupled to magnetic beads. After extensive perfusion, the remaining circulating (PE^+^ cells) cells were removed leaving behind resident CD45^+^ cells (PE^-^ cells), as well as stromal cells, including fibroblasts and cells associated with the vasculature and the lymphatic system.

Firstly, we decided to explore the broad immunological landscape in the meninges of mice at 30 dpi, when infection in the CNS is well established [9–13]. For this reason and given the scarcity of cells typically obtained from the meninges, CyTOF was used as this approach enables detection of a wide range of cell types. Our CyTOF data was composed of T cells (CD4^+^ and CD8^+^ T cells, including IFNγ^+^ subsets, NK and γδ T cells), B cells (including IgM^High^ B cells and CD11c^+^ B cells), and myeloid cells including neutrophils and macrophages (**Figs 1B–1D, S1B and S1C**), consistent with previous work [2]. Overall, we detected a significant increase in the number of CD45^+^ cells (**Fig 1E and S1 Data**) and an increased in number of the various immune subsets in the murine meninges at 30 dpi compared to naïve controls (**Fig 1F and S2 Data**), without noticeable changes in cell frequency **(Fig 1G and S3 Data**). Together, these data suggested the expansion and/or recruitment of resident immune cells into the meninges in response to infection.

**Fig 2:**
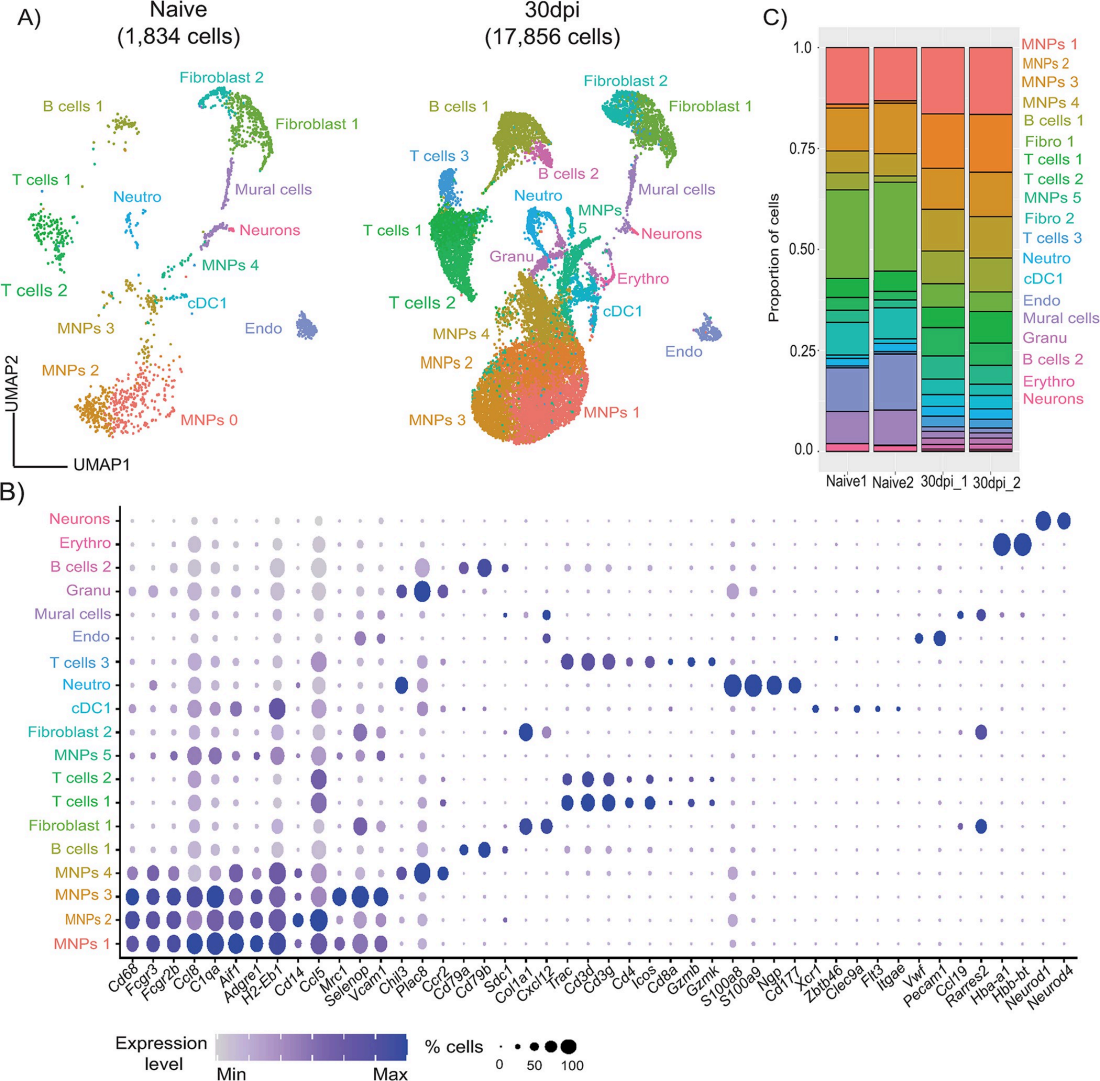
Single-cell atlas of the murine meninges during chronic *T*. *brucei* infection. **(A)** A total of 19,690 high-quality cells were used for dimensionality reduction, resulting in UMAP for the single-cell transcriptome profiling from naïve (*n* = 2 pools; 1,834 high-quality cells in total) and infected meninges (*n* = 2 pools; 17,856 high-quality cells in total). **(B)** Dot plot representing the expression levels of top marker genes used to catalogue the diversity of cell types in our single-cell dataset. **(C)** Frequency of the different cell types detected in the murine meninges analysed in this study. Supporting data in S4 Data file. MNP, mononuclear phagocyte; UMAP, uniform manifold approximation and projection.

To gain an understanding of the transcriptional responses triggered in the meninges in response to infection, and to identify potential interactions between various meningeal cells during infection, we performed single-cell RNA sequencing of meningeal preparations from mice at 30 dpi (*n* = 2 pools; 2 mice/pool) and naive controls (*n* = 2 pools; 2 mice/pool). Using this approach and after removing cells catalogued as low quality (Materials and methods), we obtained a total of 19,690 high-quality cells, from which 1,834 cells derived from naïve meninges and 17,856 cells from infected meninges, with an average of 605 genes per cell from naïve samples and 1,297 genes per cell from infected samples (**Figs 2A, S1D and S1E, and Materials and methods**). As expected, our single-cell meningeal atlas encompasses stromal and immune cells, most of which have previously been reported in the murine meninges [2,46]. Within the stromal compartment, we identified 2 populations of *Col1a1^+^* fibroblasts, *Ccl19^+^ Rarres2^+^* mural cells, and *Wvf^+^ Pecam1^+^* endothelial cells (**Fig 2A and 2B**). Within the immune compartment, we identified 5 populations of mononuclear phagocytes (MNPs 1 to 5), characterised by the expression of putative myeloid cell markers genes such as *Ccl8*, *C1qa*, *Aif1*, *Adgre1*, and *Cd14*, among others, as well as conventional DCs (cDCs; *Xcr1*, *Zbtb46*, *Clec9a*, *Flt3*, and *Itgae*) (**Fig 2A and 2B**). Additionally, we also detected T cells (*Trac*, *Cd3g*, *Cd3e*, *Cd4*, *Icos*, *Cd8a*, *Gzmb*, *Gzmk*), granulocytes, including *S100a8^+^ Ngp^+^ Cd177^+^* neutrophils, and 2 populations of B cells (*Cd79a*, *Cd79b*) that expressed markers of canonical plasma cell markers (e.g., *Sdc1*) (**Fig 2A and 2B**). Lastly, we detected a small proportion of cells (<0.5%) with high expression levels of haemoglobin (*Hba-a1*, *Hbb-bt*) and genes typically related to neurons (*Neurod1*, *Neurod4*) (**Fig 2A and 2B and S2 Table**). Notably, we observed a robust increase in the frequency of most of the cells within the immune compartment (**Fig 2C and S4 Data**) consistent with the CyTOF dataset. Together, these analyses are consistent with profound alterations in the cellular makeup of the murine meninges during chronic *T*. *brucei* infection.

### Meningeal *Rarres2^+^ Ly6a*^+^ fibroblasts acquire lymphoid tissue stroma-like properties during chronic *T*. *brucei* infection

**Fig 3:**
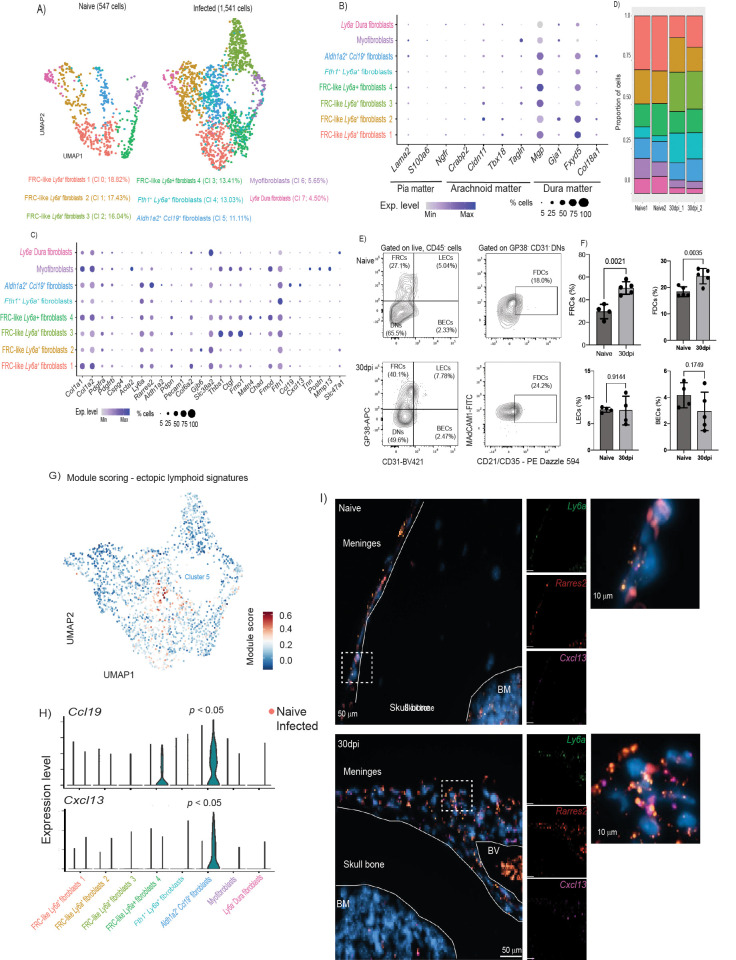
The meningeal stromal compartment acquires lymphoid-like properties and provides cues for immune cell recruitment during chronic *T*. *brucei* infection. **(A)** UMAP of 2,088 high-quality cells within the fibroblast clusters were re-analysed to identify a total 8 subclusters. **(B)** Dot plot representing the expression levels of marker genes previously reported to be enriched in fibroblasts from different meningeal layers, including dura mater, pia mater, and arachnoid mater. **(C)** Dot plot representing the expression levels of top marker genes used to catalogue the diversity of cell types in our single-cell dataset. **(D)** Frequency plot the different fibroblast clusters detected in the murine meninges in naïve and infected samples (*n* = 2 pools per experimental condition). Supporting data in S5 Data file. **(E)** Representative flow cytometry analysis of meningeal stromal cells from naïve and infected meningeal preparations (left panel) and the corresponding quantification. **(F)** Supporting data in S6 Data file. The data in all panels is expressed as mean ± SD. Data points indicate biological replicates for each panel. A parametric *T* test was employed to assess significance between experimental groups. A *p* value < 0.05 is considered significant. FRC, fibroblast reticular cells; LECs, lymphatic endothelial cells; BECs, blood endothelial cells; FDCs, follicular dendritic cells. **(G)** Module scoring for genes typically associated with lymphoid tissues. **(H)** Violin plot depicting the expression of 2 chemokines associated with lymphoid tissues such as *Ccl19* and *Cxcl13*. **(I)** smFISH demonstrating the presence of *Ly6a^+^ Rarres2*^+^ cells that express *Cxcl13* in the dura mater of the meninges of infected mice. DAPI is included as a nuclear marker. The skull bone, the BM, and the meninges are indicated. Scale bar, 50 μm. BM, bone marrow; smFISH, single-molecule fluorescent in situ hybridisation; UMAP, uniform manifold approximation and projection.

Next, we focussed on the meningeal fibroblasts. The meningeal fibroblasts are a heterogeneous cell population encompassing transcriptionally, spatially, and potentially functionally distinct units critical for meningeal immunity [47–50], but their responses to chronic protozoan infections remains to be elucidated. Therefore, we first asked whether the 3 fibroblast clusters (Fibroblast 1, Fibroblast 2, and mural cells) identified in Fig 2 contained discreet clusters of cell populations that were not resolved by the top-level clustering. After subclustering, we identified 2,088 cells (547 and 1,541 cells from naïve and infected meningeal preparations, respectively), distributed across 8 discrete subsets (**Fig 3A**). These clusters expressed marker genes putatively associated with fibroblasts from the dura mater, including *Mgp*, *Gja1*, *Fxyd5*, *and Col18a1* (**Fig 3B**) [48], but low or undetectable levels of markers proposed to be associated with arachnoid mater fibroblasts (e.g., *Clnd11*, *Tbx18*, *Tagln*) or pia mater (e.g., *Lama2*, *S100a6*, *Ngfr*) (**Fig 3B**) [48]. These observations suggested that the majority of the fibroblasts within our dataset were likely to be derived from the dura mater layer of the meninges [51]. These fibroblast clusters also expressed *Col1a1*, *Col1a2*, *Pdgfra*, and *Pdgfrb* to various degrees but lacked *Pecam1* (encoding for CD31), suggesting that these cells are of mesenchymal origin rather than endothelial [50,51] (**Fig 3C**). Furthermore, cells within clusters 0 to 5 expressed *Rarres2*, suggesting that these are meningeal pericyte-like fibroblasts [50], in addition to *Ly6a* (which encodes for the Stem Cell antigen-1, *Sca1*) (**Fig 3C**), suggesting they are likely to retain progenitor properties [50]. We thus classified these cells as *Ly6a^+^* fibroblasts (**Fig 3A–3C**). Clusters 0 to 3 also expressed *Pdpn*, which encodes for GP38, and *Col6a2*, which is recognised by the antibody ER-TR7 [52], and were thus defined as fibroblast reticular cells (FRCs)-like *Ly6a^+^* fibroblasts 1 to 4 (**Fig 3C**). Cells within cluster 4 expressed high levels of the antioxidant protein Fth1 in addition to *Rarres2* and *Ly6a* and were labelled as *Fth1^+^ Ly6a^+^* fibroblasts (**Fig 3C**). Cells within cluster 5 also expressed *Ccl19* and *Aldh1a2*, recently shown to be associated with lymphoid stroma in the milky spots [53], in addition to *Ly6a*, and were thus classified as *Aldh1a2^+^ Ccl19^+^* fibroblasts (**Fig 3C**). Cells within cluster 6 expressed high levels of *Acta2* (which encodes for α-smooth muscle actin), as well as *Tnn*, *Postn*, and *Mmp13*, and were thus classified as myofibroblasts [50]. Lastly, cells within cluster 7 expressed *Slc38a2* and *Slc47a1*, consistent with the phenotype of dura and leptomeningeal fibroblasts [54–56] recently reported to be enriched in several transporters, and were thus assigned as *Ly6a^-^* dura fibroblasts (**Fig 3C**).

We also found heterogeneous responses to infection within the meningeal fibroblast subset, including cells that display features of FRC and B cell zone reticular cells (BCRs) in response to infection. Cells within cluster 0, 1, 2, 3, and to a lesser extent cells within cluster 5 up-regulated genes associated with FRCs [57] in response to infection, including *Pdpn*, *Pdgfra*, *Pdgrfb*, *Vim*, and *Col6a3*, as well as secreted factors such as *Vefga* and *Tnfsf13b* (encoding for BAFF) (**S3A Fig**). Cells within clusters 3, 5, and 6 up-regulated genes associated with BRCs [58] during infection, including *Cxcl13* (Cluster 5), *Itga7*, *Ltbr* (cluster 5 and 6), *Madcam1* (cluster 5), *Cr2* (cluster 3 and 6), and *Tnfsf11* (encoding for RANKL) in cluster 3 (**Figs 3C and S3B**). Based on the expression pattern observed across the fibroblast subset in response to infection, we catalogued clusters 0, 1, 2, and 3 as FRC-like cells and cells within clusters 5 and 6 as BRC-like cells (**S3C Fig**). Lastly, cells within cluster 2, described as a mature population of pericytes, were exclusively detected in the infected meninges and were characterised by the expression of genes associated with blood vessel development (*Fgfr*, *Thbs1*, *Fgf2*), as well as leukocyte chemotaxis and myeloid differentiation (*Cxcl19*) (**Fig 3C and S3 Table**), suggesting de novo expansion in response to infection. Additionally, all of the FRC-like pericytes are predicted to be involved in extracellular matrix (ECM) remodelling in the meninges, including collagen and proteoglycan deposition, as well as secretion of factors involved in ECM production, as demonstrated by module scoring analysis using the MatrisomeDB database [43] (**S3D Fig**), which encompasses a curated proteomic dataset of ECM derived from a wide range of murine tissues. Consistent with these in silico predictions, we observed a consistent pattern of fibroblastic reactions and collage deposition in the dura meninges from infected mice compared to naïve controls (**S3E Fig**), further indicating an extensive meningeal ECM remodelling triggered in response to infection.

Our results so far indicate that the dura mater layer of the meninges contains a diverse population of stromal cells, including GP38^+^ and GP38^-^ stromal cells that resemble the stroma of other lymphoid tissues and ECM remodelling. Consistent with our scRNAseq data, using flow cytometry, we observed a significant expansion of GP38^+^ FRCs, and a distinctive population of MAdCAM1^+^ CD21/CD35^+^ cells indicative of the presence of FDC-like cells in the infected murine meninges compared to naïve controls (**Fig 3D–3F; Gating strategy in S2A Fig, S5 and S6 Data**), without significant changes in the lymphatic endothelial cells (LECs) or blood endothelial cells (BECs) (**Fig 3E and 3F**). Furthermore, using module scoring analysis, which allows us to assess global gene signatures associated with a gene set or pathway (in this case, ectopic lymphoid tissue signatures), we were able to identify that cells within cluster 5 were enriched for genes associated with FDC-like function and stromal lymphoid tissues, including *Ccl19 and Cxcl13*, compared to the other fibroblast clusters (**Fig 3H**), and may be derived from *Ly6a^+^* pericytes with a precursor capacity as previously reported [59,60]. Indeed, using in situ hybridisation on independent tissue sections, we were able to confirm the presence of *Ly6a^+^ Rarres2^+^* cells that expressed *Cxcl13^+^* during infection (**Fig 3I**). Together, our data demonstrate the presence of a rich diverse fibroblast population, encompassing *Ly6a^+^ Rarres2^+^* FRC-like pericytes, including *Aldh1a2^+^ Ccl19^+^* FRC-like pericytes, myofibroblasts, *Fth1^+^* fibroblasts, and perivascular dura fibroblasts. Our data also suggests that chronic *T*. *brucei* infection induces an extensive remodelling of the meningeal stroma compartment, resulting in the expansion of FRCs and FDC-like cells without significant changes in the vasculature (LECs and BECs).

### Meningeal mononuclear phagocytes are predicted to be involved in antigenic presentation and chemotaxis during chronic *T*. *brucei* infection

**Fig 4:**
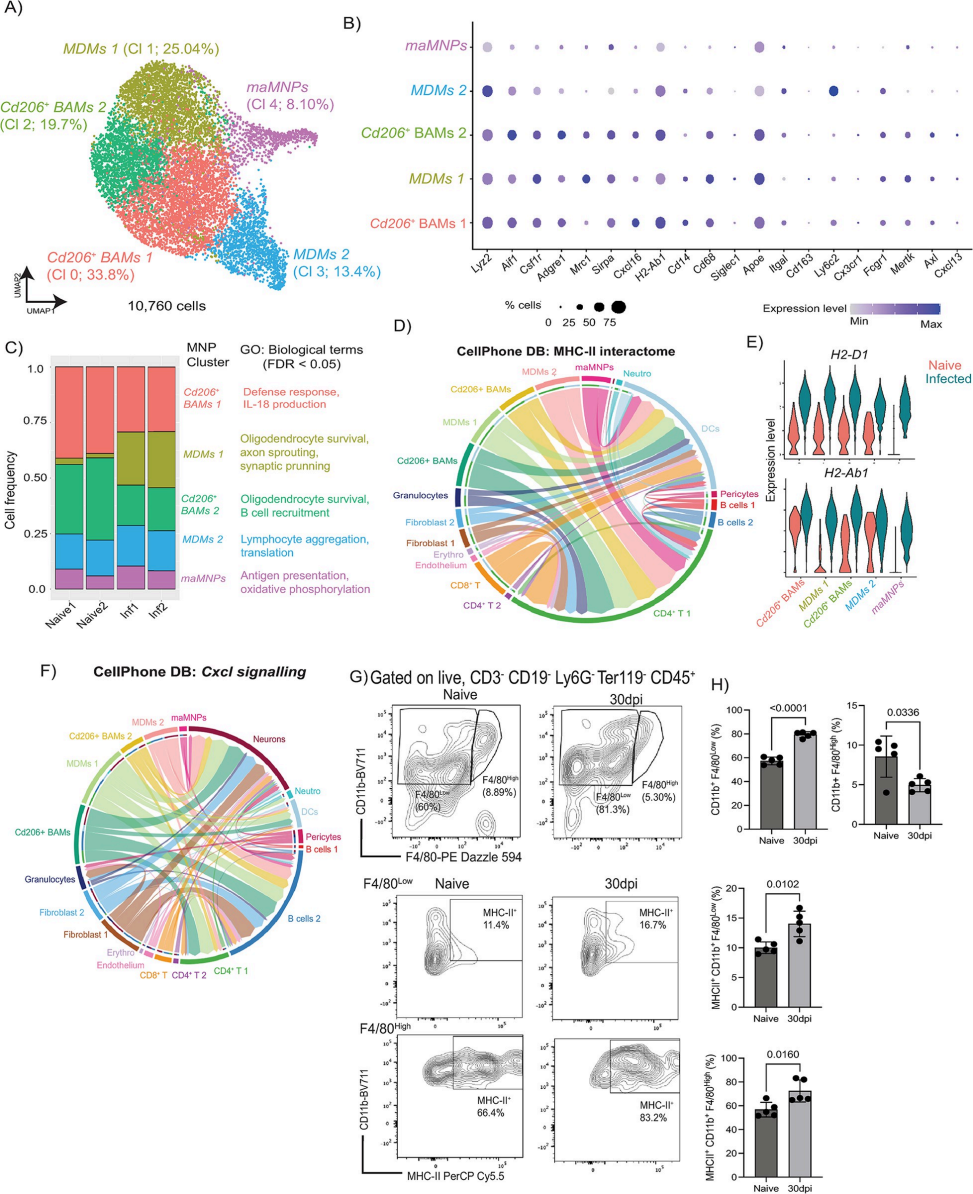
Heterogeneity of meningeal MNPs during chronic *T*. *brucei* infection. **(A)** UMAP of 10,760 high-quality MNPs from naïve (*n* = 54 cells) and infected meninges (*n* = 10,706 cells). **(B)** Expression level of top genes defining different populations of meningeal MNPs. **(C)** Frequency plot depicting the relative abundance of the 5 MNPs subclusters identified in the murine meninges during *T*. *brucei* infection. Supporting data in S7 Data file. **(D)** Cell–cell interaction network via MCH-II signalling axis. **(E)** Violin plot depicting the expression level of *H2-D1* and *H2-Ab1*, 2 of the most up-regulated MHC-II associated genes within the myeloid compartment. **(F)** Cell–cell interaction network via *Cxcl* signalling axis. **(G)** Representative flow cytometry analysis and quantification **(H)** of CD11b^+^ F4/80^High^ and F4/80^Low^ myeloid cell populations, as well as MHC-II^+^ myeloid cells, in the murine meninges in response to *T*. *brucei* infection. Data in all panels are representative from 2 independent experiments and is expressed as mean ± SD (*n* = 5 mice/experimental group). Data points indicate biological replicates for each panel. A parametric *T* test was employed to assess significance between experimental groups. A *p* value < 0.05 was considered significant. Supporting data in S8 Data file. MNP, mononuclear phagocyte; UMAP, uniform manifold approximation and projection.

The cells within the myeloid compartment in the murine meninges act as a critical first line of defence against insults and were clearly expanded during the chronic stage of the infection (**Figs 1 and 2**). To resolve the MNPs compartment in more detail, we analysed these populations individually. In total, we obtained 10,760 cells organised into 5 major clusters: cluster 0 (33.8%), cluster 1 (25.04%), cluster 2 (19.7%), cluster 3 (13.4%), and cluster 4 (8.10%) (**Fig 4A**). Since clusters 0 and 2 expressed high levels of *Mrc1* (encoding for CD206) and the anti-inflammatory molecule *Il18bp*, and *Siglec1* (encoding for CD169), we labelled these clusters as *Cd206*^+^ border-associated macrophages (BAMs) (**Fig 4B and S4 Table**). Clusters 1 and 3 contained the immune sensors *Cd14* and *Tlr2*, in addition to *Mertk*, *Adgre1*, and *Ly6c2*. We therefore labelled these clusters as monocyte-derived macrophages (MDMs). Lastly, cells within cluster 4 expressed high levels of mitochondrial-associated transcripts (e.g., *mt-Co1*, *mt-Co2*, *mt-Atp6*), in addition *Itgal*, *Sirpa*, *Cd274*, *Nfkbia*, *Sell*, and *Cd44* (**Fig 4B and S4 Table**), and were labelled as metabolically active mononuclear phagocytes (maMNPs) (**Fig 4A and 4B**). We also observed that *Cd206*^+^ BAMs and cells within the MDMs 1 cluster expressed high levels of *H2-Aa*, *Sirpa*, *Csf1r*, *Cxcl16*, and *Adgre1*, which encodes for F4/80 [61] (**Fig 4B and S4 Table**). Under homeostatic conditions, the murine meninges were dominated by *Cd206*^+^ BAMs, in agreement with previous reports [62] (**Fig 4C and S7 Data**). However, during infection, there was a significant expansion of MDMs (**Fig 4C**), suggesting that the murine meninges were populated by circulating monocytes during chronic *T*. *brucei* infection, consistent with previous reports [10]. Cell–cell interaction analyses predicted that meningeal MNPs establish significant interactions with other cell types, including T cells, via antigenic presentation (**Fig 4D–4F**), likely driving T cell activation locally, as previously proposed [2,10]. Consistent with our in silico predictions, based on the expression level of F4/80, we identified 2 populations of CD11b^+^ myeloid cells that we defined as CD11b^+^ F4/80^high^ (resembling *Cd206*^+^ BAMs and MDMs 1) and CD11b^+^ F4/80^low^ (resembling MDMs 2 and maMNPs) (**Fig 4B, 4G and 4H and S8 Data**). During infection, there was a significant increase in the frequency of CD11b^+^ F4/80^low^ MNPs, whereas the CD11b^+^ F4/80^high^ MNPs population decreased in frequency (**Fig 4G and 4H and S8 Data; Gating strategy in S2B Fig**). However, in both cases, we noted a significant increase in the expression of MHC-II in both CD11b^+^ F4/80^high^ and CD11b^+^ F4/80^low^ MNPs (**Fig 4G and 4H and S8 Data**). Together, our results indicate that the resident population of meningeal myeloid cells expand upon infection (e.g., either as a result of local myeloid proliferation or via the recruitment of monocytes to the meningeal space) likely driving both cell recruitment via chemotaxis and antigen presentation to CD4^+^ T cells. Our data are consistent with and complementary to independent reports focusing on the ontogeny and dynamics of BAMs and MNPs under homeostasis [2] and during the onset and resolution of *T*. *brucei* infection [10].

### The murine meninges contain T_FH_-like cells during chronic *T*. *brucei* infection

**Fig 5:**
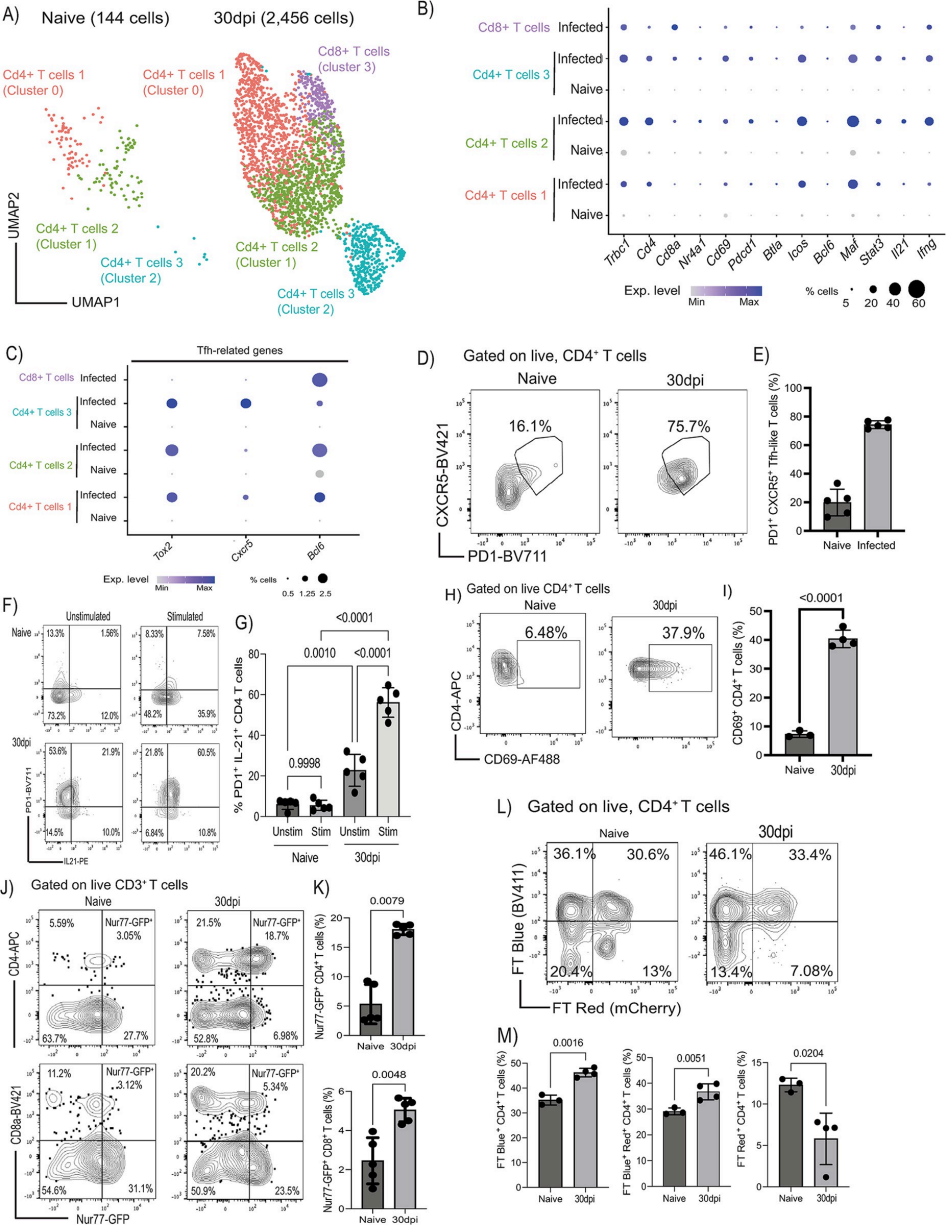
Accumulation of PD1^+^ CXCR5^+^ T_FH_-like CD4^+^ T cells in the meninges during chronic *T*. *brucei* infection. **(A)** UMAP of 1,742 high-quality T cells from naïve (*n* = 147 cells) and infected meninges (*n* = 2,691 cells). **(B)** Dot plot depicting the expression level of marker genes for all the T cell subsets identified in (**A**). The dot size corresponds to the proportion of cells expressing the marker genes, whereas the colour indicates the level of expression. **(C)** as in (B) but depicting marker genes associated with T_FH_ cells. The dot size corresponds to the proportion of cells expressing the marker genes, whereas the colour indicates the level of expression. **(D)** Representative flow cytometry analysis and quantification (**E**) the presence of CXCR5^+^ PD1^+^ CD4^+^ T cells in the murine meninges from naïve and 30 dpi (*n* = 5 mice/group). Data points indicate biological replicates for each panel. A parametric *T* test was employed to assess significance between experimental groups. A *p* value < 0.05 is considered significant. Supporting data in S9 Data file. **(F)** Ex vivo T cell activation from naïve and infected murine meninges to measure the expression of PD-1 and IL-21. Unstimulated controls are also included. **(G)** Quantification of the flow cytometry data from the ex vivo stimulation assay in (F). Data points indicate biological replicates for each panel and are representative from 2 independent experiments. A parametric *T* test was employed to assess significance between experimental groups. A *p* value < 0.05 was considered significant. Supporting data in S10 Data file. **(H)** Representative flow cytometry analysis and quantification **(I)** of the frequency of CD69^+^ CD4^+^ T cells in the murine meninges in response to infection. Data points indicate biological replicates for each panel and is representative from 2 independent experiments. A parametric *T* test was employed to assess significance between experimental groups. A *p* value < 0.05 was considered significant. Supporting data in S11 Data file. **(J)** Representative flow cytometry analysis to determine TCR engagement in CD4^+^ (top panel) and CD8^+^ (bottom panel) T cells in situ in the meninges during chronic infection with *T*. *brucei* using the Nur77^GFP^ reporter mouse line. **(K)** Quantification of the flow cytometry data from **(J).** Supporting data in S12 Data file. A *p* value < 0.05 is considered significant. Data points indicate biological replicates for each panel and are representative from 2 independent experiments. A parametric *T* test was employed to assess significance between experimental groups. A *p* value < 0.05 is considered significant. **(L)** Representative flow cytometry analysis to determine TCR engagement in CD4^+^ (top panel) in the Nur77^Tempo^ reporter mouse line. In this model, T cell activation dynamics can be discriminated between de novo (FT blue^+^) vs. historical (FT red^+^) MHC-dependent TCR engagement. **(M)** Quantification of the flow cytometry data from **(L).** Supporting data in S13 Data file. Data points indicate biological replicates for each panel and are representative from 2 independent experiments. A parametric *T* test was employed to assess significance between experimental groups. A *p* value < 0.05 was considered significant. dpi, days postinfection; FT, fluorescent timer; UMAP, uniform manifold approximation and projection.

The accumulation of inflammatory T cell subsets in the meninges has been reported in CNS infections with *T*. *brucei* [9], but their features and effector functions remain unresolved. Our top level single-cell analysis identified 3 discreet T cell clusters based on the expression of *Trac*, *Cd3e*, *Cd3g*, *Cd4*, and *Cd8a* (**Fig 2A**). To resolve the meningeal T cell compartment in more detail, we re-clustered the T cells and repeated the dimensionality reduction analysis. Within the resident meningeal T cell compartment, we identified 4 main transcriptional clusters, characterised by the expression of *Trbc1*, *Cd4* (cluster 0, 1, and 2), and *Cd8a* (cluster 3) (**Fig 5A and 5B and S5 Table**). Several of the genes observed in the CD4^+^ T cells were putatively associated with a T_FH_-like phenotype, including *Icos*, *Pdcd1* (encoding for PD-1), *Cxcr4*, *Ctla4*, *Maf*, *Nr4a1*, *Csf1*, *Tox2*, *Cxcr5*, *Bcl6*, as well as the cytokines *Ifng* and *Il21* (**Fig 5B and 5C**). To confirm this, we first examined the presence of T_FH_-like T cells in the meninges in vivo using flow cytometry. Consistent with our in silico predictions, we detected a significant increase in the frequency of resident CXCR5^+^ PD1^+^ CD4^+^ T cells in the murine meninges in response to chronic *T*. *brucei* infection compared to naïve controls (**Fig 5D and 5E and S9 Data; Gating strategy in S2C Fig**). Furthermore, ex vivo stimulation assays demonstrated that chronic *T*. *brucei* infection results in a significant expansion of meningeal PD1^+^ CD4^+^ T that express IL-21 compared to naïve controls (**Fig 5F and 5G and S10 Data**), further indicating their T_FH_-like phenotype.

Our data so far also indicate that meningeal ecosystems promote T cell activation via antigen presentation (**Fig 4D**). Indeed, both our in silico prediction and flow cytometry experiments demonstrated an expansion of CD69^+^ CD4^+^ T cells in the infected meninges compared to naïve controls (**Fig 5B, 5H and 5I and S11 Data**), strongly suggesting local activation. To examine whether meningeal T cells were activated in situ during infection, we initially utilised Nur77^GFP^ reporter mice [63]. In this model, GFP expression is used as a proxy for MHC-dependent TCR engagement resulting in T cell activation [63]. We observed a significant increase in the frequency of *Nur77*-GFP^+^ CD4^+^ and CD8^+^ T cells (**Fig 5J, 5K and S12 Data; Gating strategy in S2C Fig**), indicating local T cell activation within the murine meninges. To further resolve whether T cell activation occurs in situ, we used the newly reported *Nur77*^Tempo^ mice, a novel murine reporter line in which the expression of a fluorescent timer (FT) protein is driven by *Nur77* expression [64,65]. This model enabled the discrimination of newly activated (FT blue^+^), persistent (FT blue^+^ red^+^), and arrested (FT red^+^) T cells based on MHC-dependent TCR engagement [64,65]. We observed a significant increase in the frequency of newly activated and persistent CD4^+^ T cells and a concomitant reduction in the frequency of arrested CD4^+^ T cells in the meninges in response to infection compared to naïve controls (**Fig 5L and 5M and S13 Data**), indicating that most CD4^+^ T cells are actively partaking in the local immune response, likely via antigenic presentation. We also observed a significant increase in the frequency of newly activated meningeal CD8^+^ T cells, but a reduction in both persistent and arrested CD8^+^ T cells, perhaps indicating that the CD8^+^ T cell responses are transitory (**S4A and S4B Fig and S31 Data**). This pattern of local T cell activation was also detected in the CD69^+^ CD4^+^ T cells, in which we detected a higher frequency of newly activated CD69^+^ CD4^+^ T cells and less of arrested CD69^+^ CD4^+^ T cells (**S4C and S4D Fig and S32 Data)**, altogether indicating the meningeal T cells are newly activated in the meninges in situ. These observations are consistent with previous studies showing that CD4^+^ T cells actively patrol the meningeal landscape [2]. Taken together, these results demonstrate that the meningeal CD4^+^ T cell population undergoes newly and persistent MHC-dependent TCR engagement in the meninges, promoting local responses in situ, but the CD8^+^ T cell responses seem more transitory. These responses are likely to provide all the necessary signals for activation, likely resulting in T cell differentiation towards the observed a T_FH_-like phenotype during chronic *T*. *brucei* infection.

### The murine meninges contain plasmablasts/plasma cells and GL7^+^ CD95^+^ GC-like B cells during chronic infection

**Fig 6:**
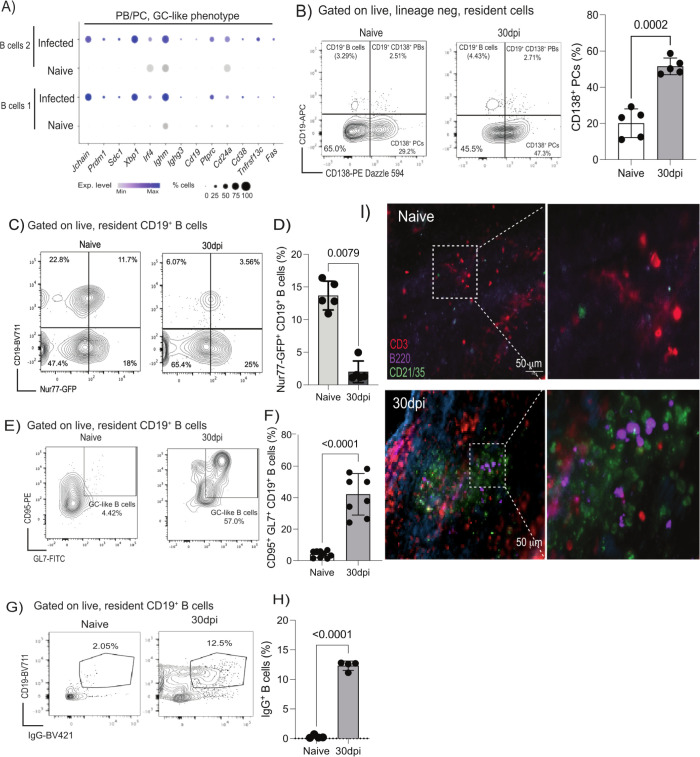
The murine meninges contain class-switched B cells in proximity to FDC-like cells. **(A)** Dot plot representing the expression level of top marker genes for the meningeal B cells, including bona fide markers of plasma cells (*Jchain*, *Prdm1*, *Sdc1*, *Xbp1*, *Ighm)* as well as activation and GC-like phenotype (*Cd24a*, *Cd38*, *Tnfrsf13c*, *Fas*). The dot size corresponds to the proportion of cells expressing the marker genes, whereas the colour indicates the level of expression. **(B)** Left panel; representative flow cytometry analysis (left panel) and quantification (right panel) to measure meningeal B cells (CD19^+^ CD138^-^ cells), plasmablasts (CD19^+^ CD138^+^), and plasma cells (CD19^-^ CD138^+^). Right panel; quantification of flow cytometry data showing the expansion of meningeal CD138^+^ plasma cells in response to infection (*n* = 5 mice/group). Data points indicate biological replicates for each panel and are representative from 2 independent experiments. A parametric *T* test was employed to assess significance between experimental groups. A *p* value < 0.05 was considered significant. Supporting data in S14 Data file. **(C)** Representative flow cytometry analysis to determine BCR engagement on meningeal B cells in situ during chronic infection with *T*. *brucei* using the *Nur77*^GFP^ reporter mouse line. **(D)** Quantification of flow cytometry data showing the reduction in the frequency of *Nur77*^GFP+^ CD19^+^ B cells in the meninges in response to infection (*n* = 5 mice/group). Data points indicate biological replicates for each panel and are representative from 2 independent experiments. A parametric *T* test was employed to assess significance between experimental groups. A *p* value < 0.05 was considered significant. Supporting data in S15 Data file. **(E)** Representative flow cytometry analysis to determine the presence of GC-like B cells in the murine meninges based on the co-expression of GL7 and CD95. **(F)** Quantification of GL7^+^ CD95^+^ GC-like CD19^+^ B cells in the meninges in response to infection (*n* = 5 mice/group). Data points indicate biological replicates for each panel and are representative from 2 independent experiments. A parametric *T* test was employed to assess significance between experimental groups. A *p* value < 0.05 was considered significant. Supporting data in S16 Data file**. (G)** Representative flow cytometry analysis to determine the presence of IgG^+^ CD19^+^ B cells in the murine meninges in response to infection. **(H)** Quantification of GL7^+^ CD95^+^ GC-like CD19^+^ B cells in the meninges in response to infection (*n* = 4 mice/group). Data points indicate biological replicates for each panel and are representative from 2 independent experiments. A parametric *T* test was employed to assess significance between experimental groups. A *p* value < 0.05 was considered significant. Supporting data in S17 Data file**. (I)** Representative imaging analysis of whole-mount meninges from naïve (left) and infected (right) of CD21/CD35^+^ FDCs (green), as well as CD3d^+^ T cells (red) and B220^+^ B cells (purple). DAPI was included as nuclear staining. Scale = 50 μm. dpi, days postinfection; FDC, follicular dendritic cell.

Previous studies have demonstrated that B cells represent a major immune population in the meninges [42,46], although their dynamics during chronic infection are not yet understood. We previously observed the expression of *Cxcl12* in dura and arachnoid meningeal fibroblasts, which is critical for the differentiation and survival of early B cells in the bone marrow [66,67]. Thus, we next explored the diversity of B cells in our dataset. The majority of meningeal B cells detected in our dataset derived from the infected meninges (1,688 cells out of 1,742 total B cells) (**Fig 2**). These cells expressed high levels of genes associated with plasmablasts and plasma cells such as such as *Jchain*, *Prdm1* (which encodes for BLIMP-1), *Sdc1* (encoding CD138), *Ighm*, and *Irf4* (**Fig 6A**). Flow cytometry experiments further confirmed that the vast majority of the meningeal B cells correspond to plasmablasts and plasma cells and to a lesser extent CD19^+^ B cells (**Fig 6B and S14 Data; Gating strategy in S2D Fig**). Some of the marker genes identified within the B cell clusters, such as *Pcna*, *Mki67*, *Ub2c*, *Ighg2*, and *Ighg3*, are typically associated with cell replication and class-switched B cells (**S2 Table**). These genes are critical for affinity maturation and class switching during GC reactions [68]. Because the transcriptional signatures observed within the B cell clusters were consistent with the presence of extrafollicular GC-like reactions, we next examined this at the protein level. We first exploited the Nur77^GFP^ reporter mouse line to measure BCR engagement within the meninges. Given that Nur77^GFP^ expression in GC B cells is proposed to be markedly reduced compared to activated B cells in vivo [69], and that *Nur77* restrains B cell clonal dominance during GC reactions [70], this reporter line can be used to examine extrafollicular GC-like reactions. In line with these studies, we detected significantly fewer GFP^+^ CD19^+^ B cells during infection compared to naïve controls (**Figs 6C, 6D and S2D and S15 Data**), implying that upon infection, the meningeal B cells undergo GC-like reactions. Intriguingly, the meningeal B cells also expressed *Cd38* and *Fas* (**Fig 6A**), similar to dark zone (GFP^low^) GC B cells [69]. Consistent with these observations, we detected a significant accumulation of GL7^+^ CD95/Fas^+^ cells within the CD19+ B cell compartment (**Fig 6E and 6F and S16 Data**), further corroborating the presence of GC-like B cells in the murine meninges. Consistent with the GC-like and the transcriptional profile, we observed an increase in the frequency of IgG^+^ CD19^+^ B cells in the murine meninges at 30 dpi compared to naïve controls (**Fig 6G and 6H and S17 Data**). In the spatial context, we observed clusters of CD3^+^ T cells, B220^+^ B cells, and CD21/35^+^ FDCs in the murine meninges that were not readily detectable in naïve animals (**Figs 6I and S5**), suggesting the presence of immunological aggregates similar to those observed in tertiary lymphoid tissues [26,27]. Together, our data indicates the presence of class-switched plasma cells/plasmablasts as well as GC-like CD19^+^ B cells in close proximity to CD3^+^ T cells and CD21^+^/CD35^+^ FDC-like cells in the murine meninges in response to *T*. *brucei* infection.

### *T*. *brucei* infection results in the accumulation of meningeal autoreactive B cells

**Fig 7:**
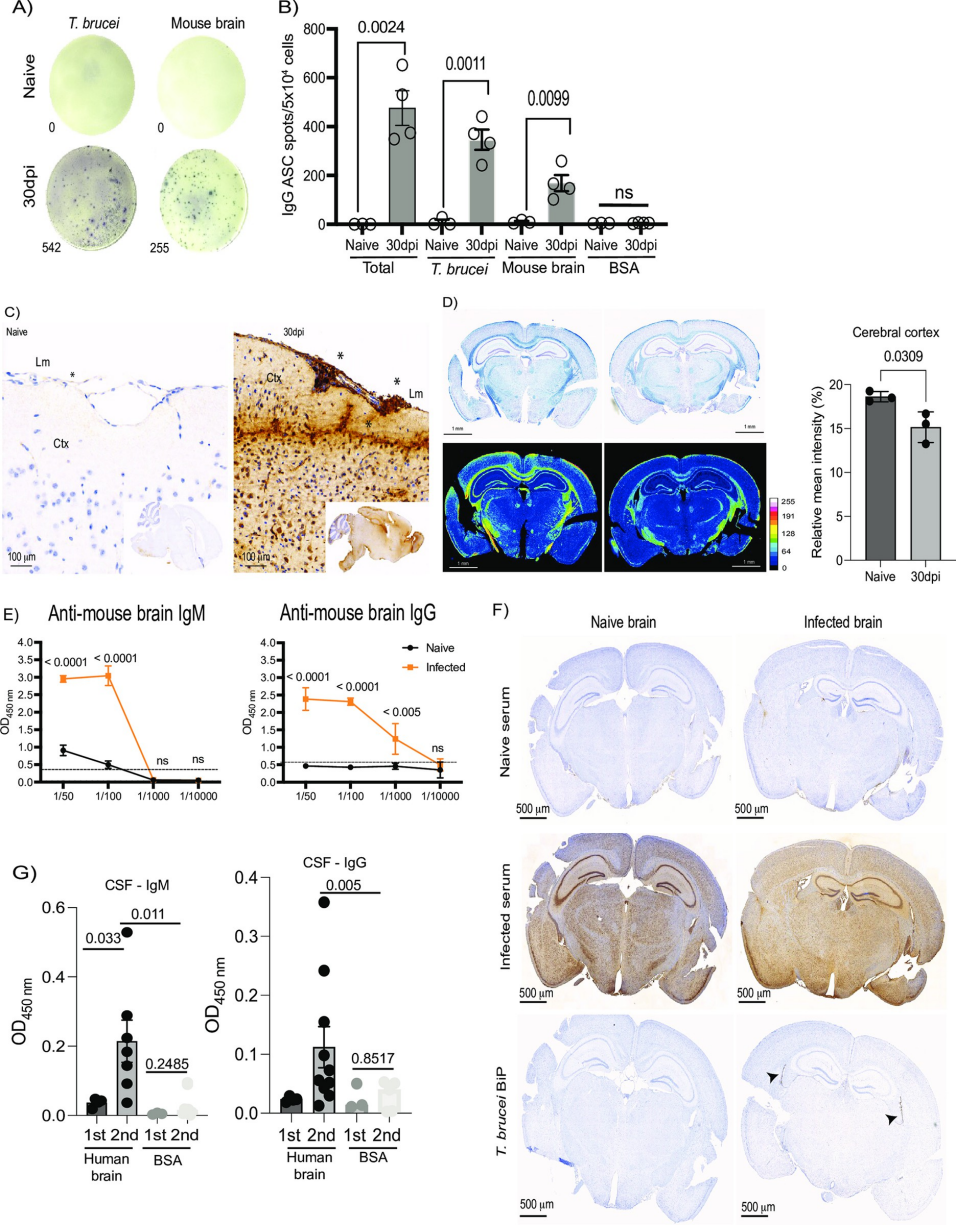
The murine meninges contain autoreactive IgG^+^ ASCs during chronic *T*. *brucei* infection. **(A)** Representative ELISpot images depicting mouse brain-specific IgG^+^ ASCs from naïve and infected murine meninges after 30 dpi with *T*. *brucei*. **(B)** Quantification of ELISpot results including total IgG^+^, *T*. *brucei*-specific IgG^+^, and mouse brain-specific IgG^+^ ASCs. Wells coated with BSA were included as negative controls. A p value < 0.05 was considered significant. Supporting data in S18 Data file**. (C)** Immunohistochemistry analysis to determine IgG^+^ deposition in the mouse brain from naïve (left) and infected (right) mouse brain sagittal sections. The sections were stained with an anti-mouse IgG antibody coupled to HRP to measure the overall distribution of IgG in the brain. The asterisks denote areas of intense IgG deposition in the leptomeningeal space, as well as in the upper layers of the cerebral cortex, exclusively detected in the infected brain. Lm, leptomeninges; Ctx, cerebral cortex. **(D)** Left panel: Representative LFB staining from naïve (left) and infected (right) animals at 30 dpi as a proxy to measure myelin. Lower panels show the tissue heatmap of the mean pixel intensity. Right panel: Percentage of demyelination, calculated here as a reduction in the average of the relative LFB intensity, was calculated from 3 independent experiments (*n* = 3–4 mice/repeat). A parametric *T* test was employed to assess significance between experimental groups. A *p* value < 0.05 was considered significant. Supporting data in S19 Data file**. (E)** Serum titters of mouse brain-specific IgM and IgG antibodies in naïve and infected samples as measured by ELISA. The dotted line represents the average of the optical density detected in naïve controls. A *p* value < 0.05 was considered significant. Supporting data in S20 Data file**. (F)** Immunohistochemistry analysis to determine the presence of circulating brain autoreactive IgG antibodies in serum from naïve (top panels) and infected animals (middle panels) in the mouse brain from naïve (left) and infected (right) mouse coronal brain sections. Staining with the *T*. *brucei*-specific protein BiP (bottom panels) is also included to highlight accumulation of parasites in the lateral ventricles (arrowheads). **(G)** ELISA analysis of human brain-autoreactive IgM and IgG antibodies in CSF from sleeping sickness patients from first stage and second stage HAT (CSF dilution 1:400). Wells coated with BSA (5 μg/ml) were included as controls. A *p* value < 0.05 was considered significant. Supporting data in S21 Data file. ASC, antibody secreting cell; CSF, cerebrospinal fluid; dpi, days postinfection; HAT, human African trypanosomiasis; LFB, Luxol fast blue.

The accumulation of meningeal B cells has been reported in several autoimmune disorders such as neuropsychiatric lupus [25] and multiple sclerosis [28] where they are responsible for the generation of autoantibodies that are linked to the pathology associated with these disorders. However, it is unclear whether chronic *T*. *brucei* infection also results in the accumulation of autoreactive B cells in the meningeal spaces. We reasoned that in addition to generating B cell clones able to generate antibodies specific to *T*. *brucei*, these local GC-like reactions taking place within the meningeal space might also result in the development of autoreactive B cells. To directly test this hypothesis, we examined the presence of meningeal resident IgG^+^ ASCs able to recognise *T*. *brucei* and mouse brain lysates using ELISpot. We observed a significant accumulation of total IgG^+^ ASCs in the murine meninges (**Fig 7A and 7B and S18 Data**), consistent with our flow cytometry data (**Fig 6G and 6H**). We also detected a significant accumulation of IgG^+^ ASCs able to recognise *T*. *brucei* and mouse brain but not BSA (**Fig 7A and 7B and S18 Data**), indicative of the presence of autoreactive ASCs in the murine meninges during infection. Interestingly, splenocytes from animals at 30 dpi or naïve controls did not contain autoreactive IgG^+^ ASCs (**S6A Fig and S33 Data**), suggesting that the mouse brain-specific autoreactive ASCs may arise locally within the meninges or within the CNS environment. Histological analysis of the corresponding murine brain sections revealed extensive IgG deposition in the infected brain compared to naïve controls, in particular in the leptomeninges and the cortex (**Fig 7C**). The IgG antibody deposition observed in the cerebral cortex in response to chronic infection was accompanied by a significant demyelination, particularly in the cerebral cortex, internal capsule, and thalamic tracts (**Figs 7D, S6B and S6C and S19 Data**). Additionally, we detected the presence of high IgM and IgG titres in the serum of infected animals able to react to mouse brain antigens compared to naïve controls (**Fig 7E and S20 Data**), further corroborating our histological and ELISpot findings. It is important to note that the binding of circulating IgG antibodies to the murine brain does not seem to be restricted to areas with high parasite accumulation (e.g., lateral ventricles) (**Fig 7F**). In humans, in the CSF of second stage *gambiense* HAT patients from North Uganda we observed significant levels of autoreactive IgM and IgG antibodies able to recognise human brain lysates, but not BSA (**Fig 7G, S21 Data and S6 Table**), consistent with our findings in experimental infections. Taken together, our data suggest that meningeal B cells undergo affinity maturation locally within the meninges or the CNS space to generate IgG^+^ ASCs directed against both *T*. *brucei* and the mouse brain (and in gambiense HAT patients), is associated with cortical and white matter demyelination, and results in autoimmunity.

### LTβ receptor signalling controls the accumulation of meningeal FDCs and autoreactive B cells during chronic *T*. *brucei* infection

**Fig 8:**
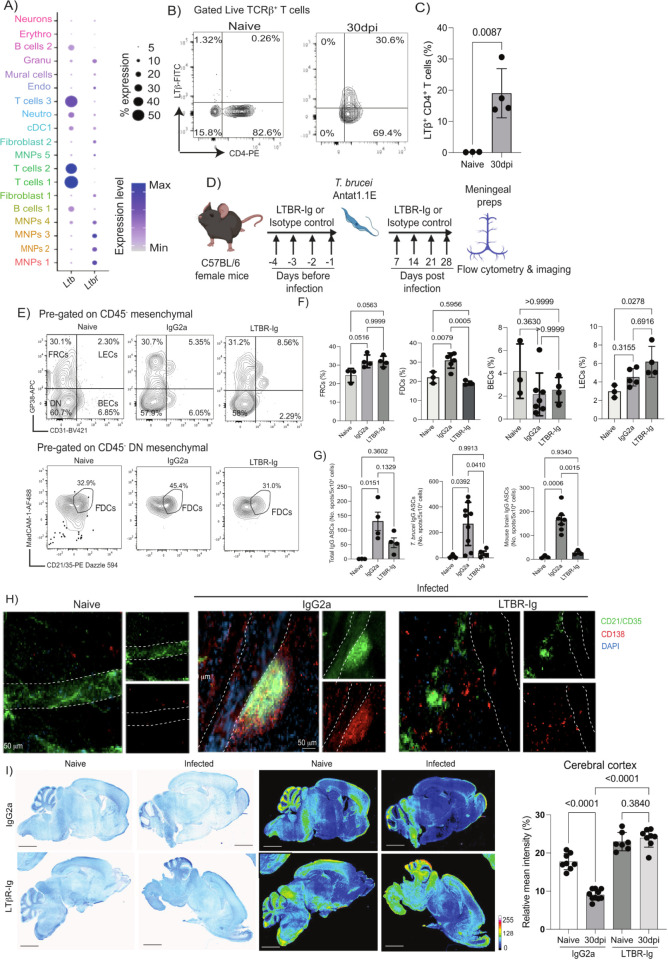
LTβ receptor signalling is critical to sustain FDC-like networks and autoreactive B cells in the murine meninges in response to *T*. *brucei* infection. **(A)** Dot plot depicting the expression level of *Ltb* and its cognate receptors *Traf2* and *Ltbr*. The dot size corresponds to the proportion of cells expressing the marker genes, whereas the colour indicates the level of expression. **(B)** Representative flow cytometry analysis depicting the expression of LTβ in CD4^+^ T cells in naïve and mice chronically infected with *T*. *brucei* (30 dpi). **(C)** Quantification of flow cytometry analysis (*n* = 4 mice/group). A *p* value < 0.05 is considered significant. Supporting data in S22 Data file**. (D)** Overview of the experimental approach applied to block LTβR signalling in vivo during chronic *T*. *brucei* infection. Images generated with BioRender. **(E)** Representative flow cytometry analysis of the murine meningeal stroma in naïve and mice chronically infected with *T*. *brucei* (30 dpi). FDC-like cells were gated from the double CD45^-^ mesenchymal cells. **(F)** Quantification of the different components of the stroma in naïve and infected meningeal preparations (*n* = 4 mice/group). A *p* value < 0.05 was considered significant. FRC, fibroblast reticular cell; LEC, lymphatic endothelial cell; BEC, blood endothelial cell; DN, double negative. Supporting data in S23 Data file**. (G)** Quantification of ELISpot results including total IgG^+^ (left panel), *T*. *brucei*-specific IgG^+^ (middle panel), and mouse brain-specific IgG^+^ ASCs (right panel) in naïve mice, mice treated with an irrelevant IgG2a antibody, and mice treated with LTBR-Ig (*n* = 4–9 mice/group). A *p* value < 0.05 was considered significant. Supporting data in S24 Data file**. H)** Representative immunofluorescence analysis of whole mount meningeal preparation labelling CD138^+^ plasma cells (red) and CD21/CD35^+^ FDC-like cells (green) in naïve or at 30 dpi. Each of the fluorescent channels is shown individually, and DAPI was included to detect cell nuclei. Scale bar = 50 μm. **(I)** Left panel: Representative LFB staining from naïve (left) and infected (right) animals at 30 dpi as a proxy to measure myelin. Middle panel: The tissue heatmap of the mean pixel intensity is also shown. Right panel: Percentage of demyelination, calculated here as a reduction in the mean grey intensity of the LFB staining, was calculated from 2 independent experiments (*n* = 4–5 mice/experiment). A parametric ANOVA with multiple comparisons was employed to assess significance between experimental groups. A *p* value < 0.05 was considered significant. Supporting data in S25 Data file. ASC, antibody secreting cell; dpi, days postinfection; FDC, follicular dendritic cell; LFB, Luxol fast blue.

LTβR signalling is critical for the formation, induction, and maintenance of lymphoid tissues under homeostasis and disease [71–73]. This process requires interactions between the LTα_1_β_2_ heterodimer and its cognate receptor LTβR to induce broad effects on FDC maintenance, promoting a favourable microenvironment promoting GC reactions on B cells [71–73]. Furthermore, expression of LTα in the meninges causes de novo ectopic lymphoid tissue formation and neurodegeneration in a model of myelin oligodendrocyte glycoprotein-induced experimental autoimmune encephalitis [74]. Our data so far indicate that the murine meninges develop ELAs that display many features of LTβ-driven lymphoid tissue formation, including the presence of FDCs like structures, T_FH_ T cells, and GC-like B cells with evidence of somatic hypermutation. Thus, we hypothesised that LTβR signalling plays a similar role in the formation of meningeal lymphoid aggregates and coordinating the meningeal responses to chronic *T*. *brucei* infection. The gene which encodes the LTβR, *Ltbr*, was expressed myeloid cells, endothelial cells, granulocytes, and fibroblasts in the meninges (**Fig 8A**), indicating that LTβR signalling may occur at multiple levels within the murine meninges. Similarly, LTβ (encoded by *Ltb*) is primarily expressed by the CD4^+^ T cell clusters and to a lesser extent by cDCs, neutrophils, CD8^+^ T cells, and B cells (**Fig 8A**). Using flow cytometry, we detected a significant increase in the frequency of CD4^+^ T cells expressing LTβ (**Fig 8B and 8C and S22 Data**), consistent with their T_FH_ phenotype [27]. Next, we investigated the role of LTβR signalling in the maintenance of local immunological responses within the meningeal stroma. For this, mice were treated prior and during *T*. *brucei* infection with a LTβR-Ig fusion protein to prevent the interaction of the ligands LTα_1_β_2_ and LIGHT (encoded by *Tnfsf14*) with LTβR (**Fig 8D**) [75]. LTβR-Ig treatment resulted in mice unable to control systemic parasitaemia as efficiently as mice treated with an irrelevant antibody or untreated mice (**S7A Fig and S34 Data**), and in a worsening in the clinical scoring (**S7B Fig and S35 Data**), mirroring previous work using *Ltb*^-/-^ mice infected with *T*. *brucei* in the context of intradermal infections [76]. Furthermore, LTβR-Ig treatment significantly impaired the expansion of meningeal FDCs (**Fig 8E and 8F and S23 Data**), and a significant accumulation of meningeal LECs compared to naïve controls, which can be attributed to changes in frequencies within other stromal compartments (**Fig 8E and 8F and S23 Data**). Using ELISpot, we observed that LTβR-Ig treatment significantly impaired the expansion of both IgM^+^ (**Figs 8G, S7C–S7E and S24 Data and S36 Data**) and IgG^+^ ASCs, including *T*. *brucei*- and mouse brain-specific ASCs (**Fig 8G and S24 Data**), consistent with a central role for LTβR signalling in the formation of B cell follicles and GCs within secondary lymphoid organs and ectopic lymphoid tissues [59,60,71,76]. Lastly, LTβR-Ig treatment significantly impaired the formation of perivascular FDC-B cell clusters (**Fig 8H**), consistent with previous reports [59,60], and prevented the cortical demyelination typically observed in response to chronic infection (**Figs 8I and S7E and S25 Data**). Together, these data demonstrate that LTβR signalling is required for stromal responses and B cell accumulation and maturation in the meninges during infection with *T*. *brucei*, further highlighting that the meninges depend on classical lymphoid tissue-associated signalling pathways to coordinate local immune responses to infections. Furthermore, the fact that LTβR-Ig treatment rescued the cortical demyelination observed in response to infection suggests that the meningeal ELAs are indeed pathogenic.

### Infection-induced autoantibodies recognise a broad range of host antigens, including myelin basic protein

**Fig 9:**
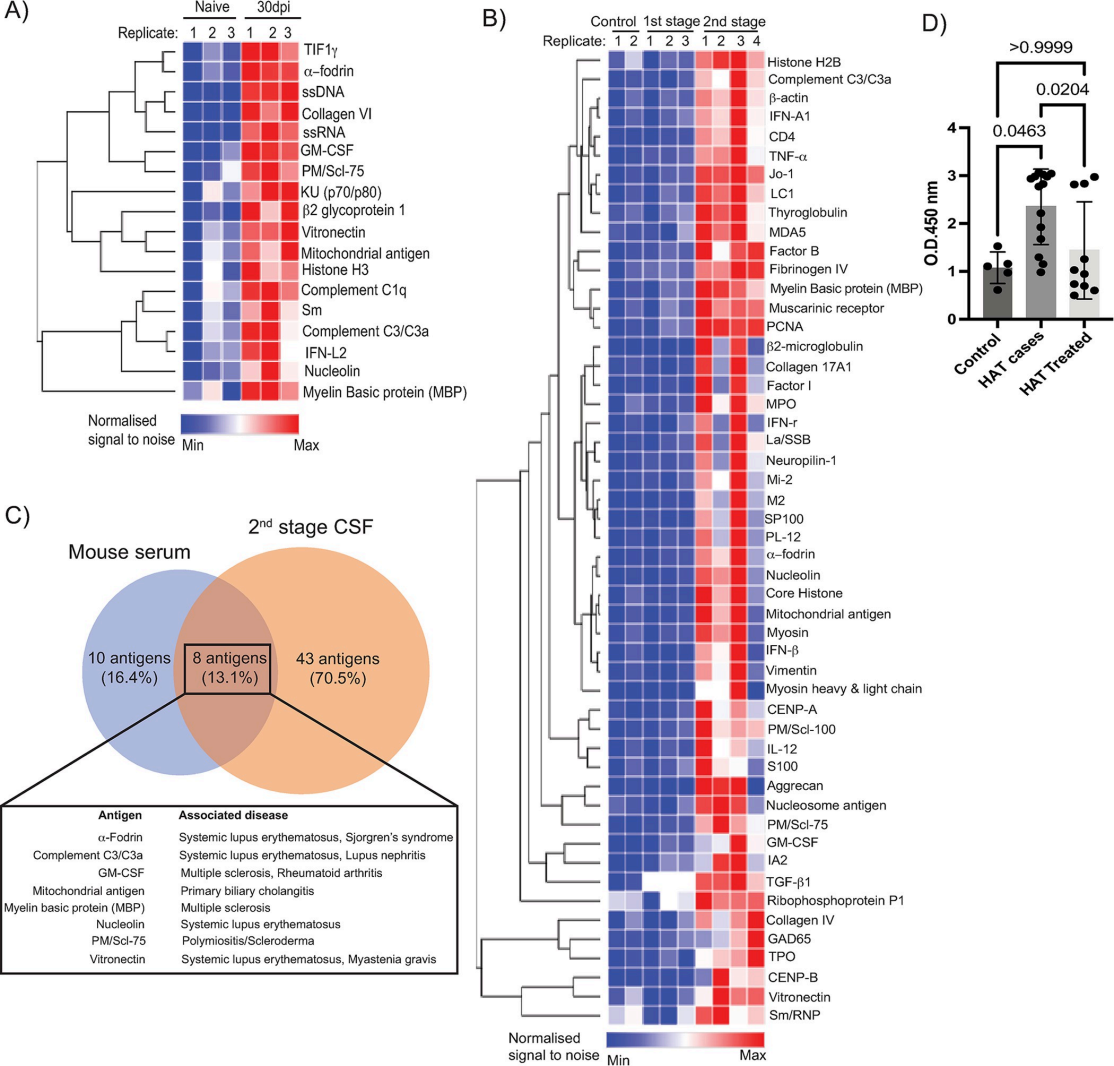
Targeted antigen screening identified shared host antigens detected by autoreactive antibodies in mouse serum and human CSF in response to *T*. *brucei* infection. **(A)** Heatmap depicting the normalised fluorescent signal-to-noise ratio for 18 antigens significantly detected by mouse serum from infected animals at 30 dpi (*n* = 3 mice) compared to naïve controls (*n* = 3 mice). The selected genes were chosen based on significant level in pairwise comparisons between naïve and infected samples using a parametric two-sided *T* test. Pairwise comparisons resulting in a *p* value < 0.05 were considered to be significant. **(B)** As in (A) but depicting a total of 51 antigens significantly and exclusively detected in the CSF from second stage sleeping sickness patients (*n* = 4 patients) compared to both first stage sleeping sickness patients (*n* = 3 patients) and healthy donors (*n* = 2 donors). **(C)** Ven diagram depicting host antigens identified in this screening that were commonly detected by autoreactive IgG antibodies in both mouse serum and human CSF, as well as those antigens that showed specie-specific responses. A table summarising the common host antigens and the disease they are often associated with is also included. **(D)** ELISA analysis to examine the presence of anti-MBP IgG autoantibodies in human serum from patients with an active *T*. *brucei* gambiense infection (“cases”) or posttreatment (“treated”), as well as healthy African controls (“controls”). A parametric ANOVA test with multiple comparison was used to estimate statistically significant pairwise comparisons. A *p* value < 0.05 was considered significant. Supporting data in S26 Data file. CSF, cerebrospinal fluid; dpi, days postinfection.

Given that our data so far indicate that chronic *T*. *brucei* infection results in the generation of autoantibodies, we next decided to examine the nature of antigens recognised by these autoantibodies. To achieve this, we employed a targeted array of 120 antigens known to be identified in autoimmune disorders, from systemic lupus erythematous to multiple sclerosis. Our data indicates that circulating IgG autoantibodies found in infected samples significantly recognised a total of 18 antigens (15% of the antigen array), including structural proteins (e.g., collagen VI, vitronectin, nucleolin, Histone H3), cytokines (e.g., GM-CSF), components of the complement system (e.g., C3, C1q), intracellular antigens (e.g., ssDNA, ssRNA, mitochondrial antigen), and most importantly MBP (**Figs 9A and S8A and S7 Table**). To further understand whether the same pattern of autoreactive antibodies is observed in sleeping sickness patients, we screened CSF samples collected from patients during the first (haemolymphatic) stage and second (meningoencephalitic) stage. As observed in mice, our results highlighted a broad range of host antigens recognised by IgG autoantibodies in the CSF exclusively detected during the second stage of the disease (**Figs 9B and S8B and S7 Table**). More specifically, we detected reactivity against 51 antigens (42.5% of the antigen array), including several structural proteins, cytokines (e.g., TGFβ1, TNFα, IL-12, TPO), intracellular antigens (e.g., histones, nucleosome-related proteins, mitochondrial antigen), structural proteins (e.g., collagens, vitronectin), among others (**Fig 9B**). Interestingly, as observed in mice, we also detected the presence of autoantigens able to bind host proteins associated with either parasite control or pathology, such as proteins of the complement system (e.g., C1q, C3a) and nervous system-associated proteins (e.g., MBP and muscarinic receptor) (**Fig 9B**). Indeed, a total of 8 antigens (13.1% of the antigen array) were commonly identified by autoreactive antibodies in infected mouse serum and human CSF from second stage sleeping sickness patients, which are known to be diagnostic markers of autoimmune disorders such as systemic lupus erythematosus, Sjogren’s syndrome, scleroderma, rheumatoid arthritis, and multiple sclerosis [77–79] (**Fig 9C**). To further validate our findings, we examined the presence of circulating antibodies against MBP in an independent cohort of sleeping sickness patients from DRC that included both patients with an active infection (“cases”) and samples obtained from patients posttreatment (“treated) (**Fig 9D and S26 Data**). Consistent with the data obtained from CSF biopsies, we observed that sleeping sickness patients with an active infection have significantly higher titres of serum IgG against MBP compared to healthy African controls (**Fig 9D and S26 Data**). Interestingly, most of the samples obtained from patients posttreatment display basal levels of anti-MBP antibody titres and show no significant differences with healthy African controls, suggesting that treatment with antiparasitic chemotherapy prevents the accumulation of anti-MBP autoantibodies in humans. However, we noted that 30% of the treated patients maintained higher titres of anti-MBP antibodies in circulation, which might be due to: (i) failure to effectively clear parasites posttreatment and thus considered to be relapsing cases; (ii) the presence of memory B cells that sustain anti-MBP autoantibody secretion; (iii) or a combination of both. Taken together, our mid-throughput targeted screening identified a myriad of host antigens recognised by infection-induced autoantibodies in both chronically infected mice and second stage sleeping sickness patients, potentially indicative of a complex autoimmune disorder affecting several organs including the CNS.

### The accumulation of meningeal GL7^+^ CD95^+^ GC-like B cells and autoreactive antibodies depends upon parasite accumulation in the CNS

**Fig 10:**
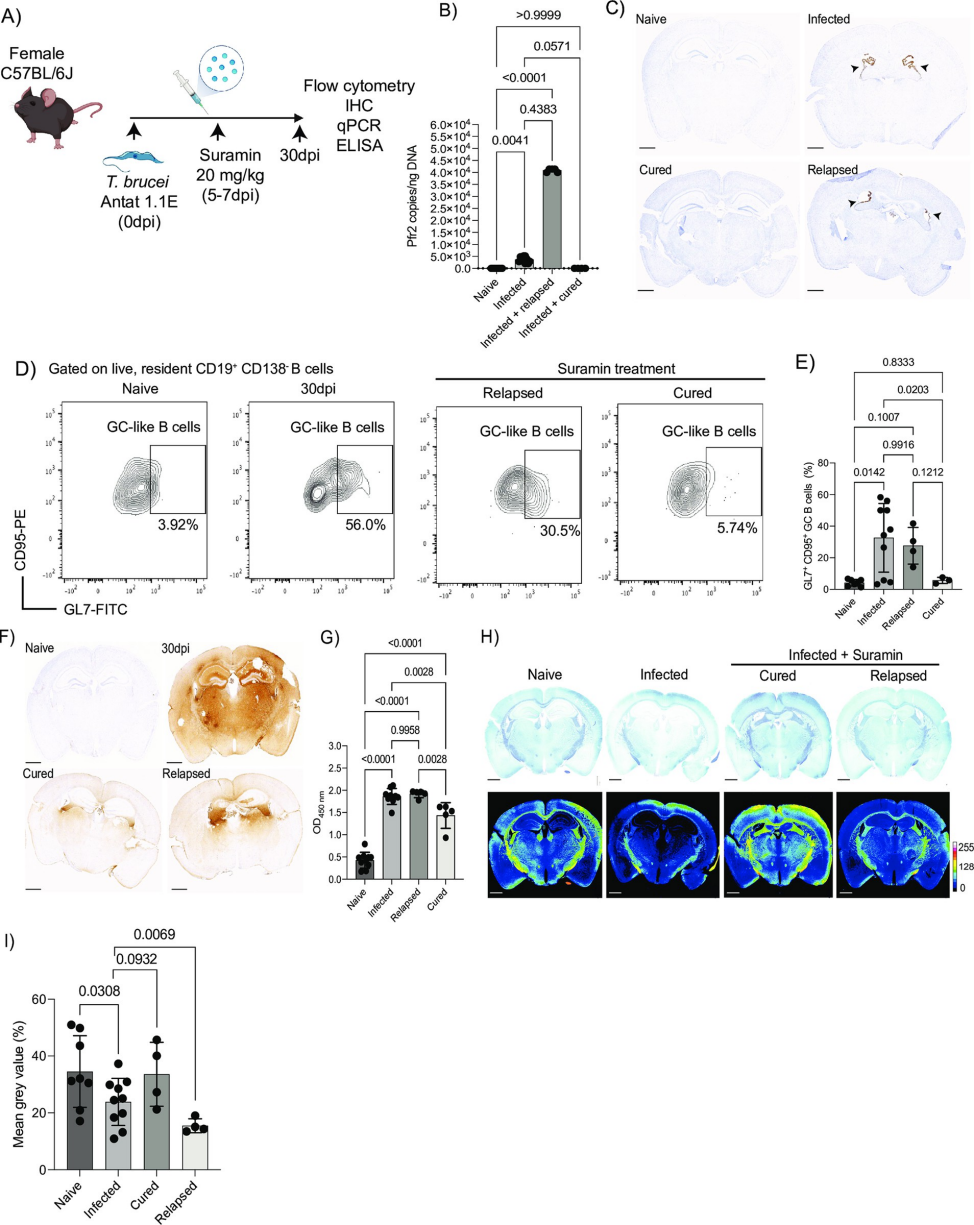
Suramin treatment prevents the expansion of GL7^+^ CD95^+^ GC-like B cells and the IgG deposition in the mouse brain. **(A)** Overview of the experimental approach applied to prevent the CNS stage of the disease using suramin. Images generated with BioRender. **(B)** Estimation of *T*. *brucei* burden in the murine brain using RT-PCR analysis to detect the parasite-specific *Pfr2* gene in naïve brain specimens and infected but untreated animals (*n* = 5 mice/group), as well as cured (*n* = 4 mice) and relapsing animals (*n* = 4 mice). These data are representative of 2 independent experiments. A parametric ANOVA test with multiple comparison was used to estimate statistically significant pairwise comparisons. A *p* value < 0.05 was considered significant. Supporting data in S27 Data file**. (C)** Immunohistochemistry staining of the *T*. *brucei*-specific protein BiP in brain sections from the same experimental groups as in (B). **(D)** Representative flow cytometry analysis of GL7^+^ CD95^+^ GC-like CD19^+^ B cells in the murine meninges from the same experimental groups as in (B). The gating strategy to identify meningeal B cells is shown in S2C Fig. **(E)** Quantification of GL7^+^ CD95^+^ GC-like CD19^+^ B cells in the murine meninges from the same groups as in (B). A parametric ANOVA test with multiple comparison was used to estimate statistically significant pairwise comparisons. A *p* value < 0.05 was considered significant. Supporting data in S28 Data file. **(F)** Representative immunohistochemistry micrographs comparing IgG^+^ deposition in brains from naïve mice and infected but untreated mice, as well as cured and relapsed mice post suramin treatment. Images are representative from 2 independent experiments. Scale bar = 1 mm. **(G)** ELISA test to determine serum IgG titters of mouse brain-specific autoantibodies in the experimental groups in (B). Data are representative from 2 independent experiments. A parametric ANOVA test with multiple comparison was used to estimate statistically significant pairwise comparisons. A *p* value < 0.05 was considered significant. Supporting data in S29 Data file. **(H)** Upper panel: Representative LFB staining as a proxy to measure myelin in brain specimens from naïve and infected mice, as well as infected mice treated with suramin, including relapsed and cured animals. Lower panel: Tissue heatmap representing the MGV for the LFB staining. The calibration bar was set up so that the lowest level is 0 and the maximum MGV is 255. Scale bar = 1 mm. **(I)** Percentage of demyelination, calculated here as a reduction in the mean grey intensity of the LFB staining, was calculated from 2 independent experiments (*n* = 4–10 mice/experiment). Parametric pairwise comparisons using one-sided *T* test was employed to assess significance between experimental groups. A *p* value < 0.05 was considered significant. Supporting data in S30 Data file. CNS, central nervous system; LFB, Luxol fast blue; MGV, mean grey value.

Our data so far demonstrate that chronic *T*. *brucei* infection results in the accumulation of autoreactive B cells that display a GL7^+^ CD95^+^ GC-like phenotype, likely resulting in the generation of autoreactive antibodies and subsequent local IgG deposition in the brain. We also identified MBP, a highly abundant CNS protein, to be one of the host antigens recognised by these infection-induced IgG autoantibodies in both mice and humans during chronic infections, potentially explaining the local antibody deposition observed in the brain in our histological analyses. Given that at least 30% of second stage sleeping sickness patients displayed elevated levels of anti-MBP autoantibodies in circulation posttreatment, likely as a result of treatment failure, we next decided to explore whether suramin treatment, used in experimental infections to clear *T*. *brucei* infections [18,80], prevented the accumulation of GL7^+^ CD95^+^ GC-like phenotype and IgG deposition in the brain. In other words, whether an active CNS colonisation is necessary to trigger local B cell responses. We tried several treatment strategies based on recent studies [18,80], the majority of which resulted in mice relapsing to the infection. This was particularly evident when treatment was started after 14 dpi. In our hands, the most effective treatment regime consisted of 3 consecutive doses of suramin (20 mg/kg) i.p. at 5, 6, and 7 dpi, consistent with previous studies [80] (**Fig 10A**). Using this model, we observed approximately 50% of mice relapsing and approximately 50% of the animals completely clearing the disease, as determined by qPCR against the *T*. *brucei*-specific gene *Pfr2* used here as a proxy to quantify parasite tissue burden, alongside immunohistochemistry staining against the *T*. *brucei*-specific antigen BiP (**Fig 10B and 10C and S27 Data**). Interestingly, in the relapsing animals, we noted a significantly higher parasite burden in the brain compared to infected but untreated controls. Using flow cytometry, we detected a significant expansion of GL7^+^ CD95^+^ GC-like B cells in the meninges of infected animals that remained high in the relapsing animals (**Fig 10D and 10E and S28 Data**). However, in cured mice, the frequency of GL7^+^ CD95^+^ GC-like B cells in the meninges returned to basal levels (**Fig 10D and 10E**). Furthermore, we observed a reduction in the IgG deposition in the brain of cured mice (**Fig 10F**), reduced serum antibody titres of anti-brain IgG autoantibodies in cured mice compared to infected or relapsing animals (**Fig 10G and S29 Data**), and less cortical demyelination in cured mice compared to infected and relapsing animals (**Fig 10H and 10I and S30 Data**), indicating that an active CNS infection is required to induce the pathological antibody responses and cortical demyelination observed in response to chronic infection. However, it is worth noting that cured mice still showed signs of antibody deposition and serum levels of anti-brain IgG autoantibodies, albeit to a lesser extent to infected or relapsing animals. Taken together, our results suggest that the presence of parasites in the CNS either directly or indirectly promotes the expansion of meningeal GL7^+^ CD95^+^ GC-like B cells and antibody deposition in the brain during chronic infection.

## Discussion

Here, we set out to characterise the local immune responses taking place within the murine meninges in response to chronic infection with *T*. *brucei*. Our results demonstrate that the murine meninges are dynamic structures able to support a wide range of immune responses that are triggered upon infection with *T*. *brucei*, resulting in the acquisition of ectopic lymphoid tissue-like signatures including the development of FDC-like cells, T_FH_ cells, and GC-like B cells undergoing class switching. We also showed that during *T*. *brucei* infection, the murine meninges also harbour a distinctive population of autoreactive B cells that generate IgG^+^ antibodies able to bind parasite and murine brain antigens, including MBP. Furthermore, we demonstrated that the rearrangement of the meningeal stroma, as well as the accumulation of autoreactive B cells, depend upon LTβR signalling, consistent with its lymphoid tissue properties. Lastly, we demonstrated the presence of IgG^+^ antibodies in the CSF of second stage gambiense HAT patients (when the parasites accumulate in the meninges and CNS) able to recognise human brain lysates and MBP, indicating that the observations using experimental infections are likely to be conserved in humans.

Focussing on the stroma, we identified several populations of meningeal fibroblasts, mostly derived from the dura mater layer of the meninges that express bona fide markers of mesenchymal precursor cells (*Ly6a^+^*). Interestingly, this population of meningeal fibroblasts shares many transcriptional features with omental *Aldh1a2^+^* FRCs that are known to play a critical role in modulating lymphocyte recruitment and local immune responses in the peritoneum [53]. It is tempting to speculate that there exists a predefined populations of fibroblasts with precursor capacity (e.g., Ly6a/Sca1^+^) in several body cavities, including the meninges, that are able to quickly sense and adapt to inflammatory responses to efficiently coordinate local immune responses [81–83]. In this context, it is plausible that the population of lymphoid stromal cells residing in the dura layer of the meninges sense the presence of *T*. *brucei* (e.g., via TLR or cytokine signalling) to promote local immunological responses, as recently proposed [84]. We propose that these populations meningeal *Ly6a^+^* fibroblast precursors could adapt to chronic inflammatory conditions, resulting in the development of stromal structures required to sustain long-lasting immunological responses in situ, including FRC- and FDC-like cells, as well as de novo angiogenesis. These observations are consistent with the idea that chronic neuroinflammation results in lymphangionesis in the CNS [85]. All of these populations participate in changes in the ECM during infection, including collagen and proteoglycan deposition and regulation of the ECM, highlighting the extensive ECM remodelling taking place in the meninges during chronic infection. The ontogeny of the meningeal FDC-like cells that we detected under chronic infection with *T*. *brucei*, and whether they play an active role in the neuroinflammatory process in this infection setting [86], requires further investigation. Nevertheless, to our knowledge, this is the first report characterising the response of meningeal fibroblasts to chronic infections with a protozoan parasite, which has important implications for understanding how dynamic and adaptable the meningeal stroma is under chronic inflammatory processes.

Additionally, we predicted that meningeal MNPs are involved in chemotaxis and antigen presentation to CD4^+^ T cells. Consistent with recent studies characterising the dynamics of myeloid cells within the brain borders in response to *T*. *brucei* infection [9,10], we identified several subsets of MNPs and conventional DCs that play a wide range of roles, from inflammatory responses to chemotaxis and antigenic presentation. Overall, all these myeloid subsets might offer a first line of defence against incoming parasites, and together with the meningeal stroma, might promote the recruitment and activation of adaptive immune cells required to support an efficient local immune response. It also remains to be determined whether the lymphatic structures present within the meninges play an active role in the induction of ectopic lymphoid tissues during chronic *T*. *brucei* infection.

We also detected an accumulation of autoreactive B cells able to produce high-affinity antibodies against both *T*. *brucei* and the mouse brain, resembling the pathology observed under autoimmune neurological disorders including MS [87]. Previous work has demonstrated the presence of autoreactive antibodies in HAT patients and during experimental infections [88–92], some of which are able to recognise a wide variety of heat shock proteins and ribosomal proteins [88,90] that might be evolutionarily conserved between parasites and host. However, the local generation of autoreactive B cells in the murine meninges able to recognise murine brain lysates in response to chronic infection with *T*. *brucei* has not been reported before. These observations indicate that the local immunological responses in the meninges could result in the (uncontrolled) production of autoreactive antibodies, explaining their presence in the CSF of second stage *gambiense* HAT patients as shown in this study. Nevertheless, the mechanisms resulting in the development of local autoantibodies remains to be determined.

Based on our dataset and in vivo experimental approaches, we conclude that the formation of meningeal ELAs during chronic *T*. *brucei* infections relies on LTβR signalling. This in turn is likely to supports the expansion of meningeal FDC-like fibroblasts and the accumulation of GC-like autoreactive B cell clones and brain deposition of high-affinity antibodies, potentially aided by the local activation and development of CXCR5^+^ PD1^+^ T_FH_-like CD4^+^ T cells expressing high levels of *Il21*. In this context, our data demonstrate that chronic meningeal inflammation leads to the formation of plasma cells/FDC-like cell/CD3^+^ T cell aggregates, resembling those within the B cell follicle in lymph nodes and spleen but lacking their typical microarchitecture, to sustain the production of high-affinity antibodies locally. It is important to note that the majority of the cells within the B cell compartment were assigned as IgM^+^ and IgG^+^ plasma cells as shown before [2,42,46,93,94], although their composition (IgA^+^ versus IgM^+^ and/or IgG^+^) differs slightly from previous elegant work describing the diversity of meningeal B cells compartment during fungal infection [46]. Interestingly, in an experimental autoimmune encephalomyelitis (EAE) model, the frequency of *Igha*^+^ B cells found in the homeostatic dura mater decreased significantly followed by a significant expansion of *Ighm* and *Ighg* expression in B cells during inflammation [93], in a process similar to the results presented in this study. These differences might be attributed to technical differences between studies (e.g., depth of coverage) or biological differences due to intrinsic differences between disease conditions. It is important to note that, in addition to the autoreactive IgG^+^ ASCs residing in the meninges and identified by ELISpot, vascular leakage (allowing the passage of IgG through the blood–brain barrier) reported in this model [95–97] may also contribute to the IgG deposition detected within the brain. However, at present we cannot directly assess the relative contribution of each process separately. Irrespective of the routes by which autoreactive antibodies reach the meningeal barrier and/or brain parenchyma, further work is required to identify whether they arise as a result of molecular mimicry (e.g., shared epitopes between *T*. *brucei* and mice) or via bystander activation (e.g., continues TLR stimulation on B cells [98,99]). It remains unclear, however, whether the emergence of autoreactive B cells depends upon T cell-mediated responses (antigen-specific) or whether it results from T cell-independent processes (e.g., polyclonal activation, antigen-independent). In the context of T cell-dependent responses, future work is required to determine the nature of the antigens driving such specific responses, as well as the precise ontogeny of T_FH_ T cells (e.g., derived from T_H_17 T cells as recently proposed [100]) to the generation of high-affinity autoreactive antibodies in the context of chronic infections remains to be delineated in more detail. It is likely that brain antigens such as MBP might be one (of several) antigen driving T cell-specific responses, in a similar process to experimental autoimmune encephalitis [101,102].

The histological features related to antibody deposition in the meningeal and cortical spaces during chronic *T*. *brucei* infection are associated with cortical and white matter demyelination, which are reminiscent of the histopathological features observed in MS and other neurological autoimmune disorders [103–105]. However, it is unclear whether the cortical pathology observed in our infection model results in primary (B cells generating antibodies against myelin) or secondary demyelination, as a result of neuronal death. Importantly, *Aid*^-/-^ mice, in which B cells are unable to undergo affinity maturation, are better at controlling *T*. *brucei* infections due to an increase in circulating (low affinity) IgM, suggesting that class-switching might indeed be an unfavourable process for the host [106], both in terms of parasite control and tissue immunopathology. Based on our data, local B cell affinity maturation and class-switching results in autoimmunity. In this scenario, it is tempting to speculate that most of the neuropathological features associated with chronic *T*. *brucei* infection derive from a disruption in peripheral tolerance resulting in maladaptive antibody responses, as recently demonstrated in variable immune deficiency patients with autoimmune manifestations [107]. Further studies are required to determine the type of antigens detected by the autoreactive antibodies generated specifically in the meninges, and to determine whether they share epitopes with *T*. *brucei* antigens due to molecular mimicry, as reported for EBV-induced MS [87]. Similarly, it is important to determine if B cell depletion strategies (e.g., B cell depletion approaches [108,109], including treatment with anti-CD20 treatment [110]) or chemotherapy interventions to treat the infection ameliorate disease progression, meningeal inflammation, and cortical pathology during chronic *T*. *brucei* infection, similar to MS [109]. Lastly, our observations in both experimental infections and human studies indicate that sleeping sickness results in an autoimmune disorder affecting the CNS (and likely other organs), but it remains unclear whether these autoimmune disorders have an impact of sleep, contributing to the known sleep disruptions caused by this parasitic infection. In this regard, sleep disturbances are commonly reported in patients with autoimmune encephalitis [111–113], and in narcoleptic patients [114,115], potentially supporting a link between these pathologies. However, this remains to be further tested and the mechanisms elucidated.

Together, our data provide a novel perspective for understanding the cellular and molecular mediators that lead to the development of autoimmunity during chronic *T*. *brucei* infection. Furthermore, our results support the notion that the meningeal spaces are dynamic structures able to support a wide range of immunological responses, including those resulting in pathological outcomes such as autoreactive antibody deposition at the brain borders. In this context, we propose that experimental infections with African trypanosomes can be exploited to address basic questions regarding infection-induced autoimmunity and brain pathology, which could be leveraged for the treatment of complex neurological disorders of unknown aetiology such as MS in addition to the meningoencephalitic stage of sleeping sickness. Our results also highlight that chronic sleeping sickness in patients also results in the accumulation of autoreactive antibodies in the CNS, potentially driving pathology even after antiparasitic chemotherapy. In this context, it becomes clear that a better understanding of the sequalae of the infection in human and animal health is critical but remains unsolved.

## Supporting information

S1 Fig Quality control measurements of the murine single-cell CyTOF and transcriptomics dataset. **(A)** Representative flow cytometry analysis of the input and flowthrough for the removal of circulating CD45^+^ immune cells using magnetic sorting. **(B)** Identification of CD45^+^ cells in the CyTOF murine meninges dataset. **(C)** Uniform manifold approximation and projection (UMAP) of the CyTOF dataset from samples (left panel; BC10-BC15: naïve; BC11-BC20: 30 dpi) experimental groups (right panel) after CD45^+^ cell clustering. **(D)** Number of unique molecular identifies (UMIs), genes, mitochondrial reads, and library complexity (Log10 UMIs/gene) after applying filtering parameters. **(E)** Clustree output representing the relationship between different cell clusters at various levels of resolution using the function *FindClusters*.
(EPS)

S2 Fig Gating strategies for flow cytometry analysis. Gating strategies to identification stromal cells (**A**), resident myeloid cells (**B**), resident CD4^+^ and CD8^+^ αβ T cells (**C**), and B cells/plasma cells (**D**).
(EPS)

S3 Fig Expression of lymphoid stromal cells marker genes within the dura fibroblasts. **(A) Left:** Expression level of Marker genes typically associated with fibroblast reticular cells (FRCs). **Right:** Expression level of genes encoding for secreted factors resealed by FRCs. **(B)** Marker genes moderately (left) or lowly (right) expressed typically associated with B zone reticular cells (BRC). **(C)** Broad classification of the various fibroblasts clusters as FRC- or BRC-like clusters based on the marker genes shown in (A) and (B). In all cases, the size of the dot represents the proportion of cells expressing the markers indicated in each plot. **(D)** Feature plots depicting the results from module scoring of the different categories within the MatrisomeDB including collagen and proteoglycan production and deposition and secreted factors. **(E)** Masson’s trichrome staining depicting collagen deposition (blue) and keratin (pink) in sagittal skull sections containing dura meninges from naïve and infected animals. Scale bar = 50 μm.
(EPS)

S4 Fig Local T cell activation in the meninges in response to *T*. *brucei* infection using the *Nur77*^Tempo^ reporter mice. **(A)** Representative flow cytometry analysis to determine TCR engagement in CD8^+^ T cells in the *Nur77*^Tempo^ reporter mouse line. In this model, T cell activation dynamics can be discriminated between de novo (FT blue^+^) versus historical (FT red^+^) MHC-dependent TCR engagement. **(B)** Quantification of the flow cytometry data from **(A)** focusing exclusively on FT blue^+^ or FT red^+^ CD8^+^ T cells. Data points indicate biological replicates for each panel and are representative from 2 independent experiments. A two-sided, parametric *T* test was employed to assess significance between experimental groups. A *p* value < 0.05 was considered significant. Supporting data in S31 Data file. **(C)** Representative flow cytometry analysis to determine TCR engagement in CD69^+^ CD4^+^ T cells in the *Nur77*^Tempo^ reporter mouse line. **(D)** Quantification of the flow cytometry data from **(A)** focusing exclusively on FT blue^+^ or FT red^+^ CD69^+^ CD4^+^ T cells. Data points indicate biological replicates for each panel and are representative from 2 independent experiments. A two-sided, parametric *T* test was employed to assess significance between experimental groups. A *p* value < 0.05 was considered significant. Supporting data in S32 Data file.
(EPS)

S5 Fig Meningeal lymphoid-like aggregates in response to *T*. *brucei* infection. Additional imaging analysis of whole-mount meninges from naïve (left) and infected (right) of CD21/CD35^+^ follicular dendritic cells (green), as well as CD3d^+^ T cells (red) and B220^+^ B cells (purple). The data correspond to data obtained from 2 independent meningeal preparations; Replicate 1 and 2 shown in top and bottom panels, respectively. DAPI was included as nuclear staining. Scale = 100 μm.
(EPS)

S6 Fig Chronic *T*. *brucei* infection induces demyelination in the CNS. **(A) Left:** Representative ELISpot images depicting mouse brain-specific IgG^+^ ASCs from naïve and infected murine meninges and splenocytes after 30 dpi with *T*. *brucei*. **Right:** Quantification of ELISpot results from mouse brain-specific IgG^+^ ASCs in meningeal preps and splenocytes (*n* = 4 mice/group). A *p* value < 0.05 is considered significant. Supporting data in S33 Data file. Luxol Fast blue staining to determine myelination in naïve (**B**) and infected (**C**) coronal brain sections. Insets how areas of the cortex (Ctx), internal capsule (IC), and thalamus (Th).
(EPS)

S7 Fig LTβR signalling blockade results in uncontrolled parasitaemia and IgM^+^ B cell accumulation in the meninges. Parasitaemia (**A**) and clinical scoring (**B**) of *T*. *brucei*-infected mice treated with the LTβR-Ig fusion protein (blue line). *T*. *brucei*-infected mice alone (green line) or infected mice treated with an irrelevant IgG2a antibody (orange line) were used as controls. For parasitaemia, an ANOVA test with multiple comparisons was conducted. For clinical scoring, pairwise comparisons were conducted using a nonparametric *T* test. In all cases, a *p* value < 0.05 was considered significant. Supporting data in S34 and S35 Data file for parasitaemia and clinical scoring, respectively. Representative ELISpot results for meningeal IgM^+^ (**C**) and IgG^+^ (**D**) antibody secreting cells (ASCs), including total ASCs (top panel), *T*. *brucei*-specific ASCs (middle panel), and mouse brain-specific ASCs (bottom panel). The number of spots detected by the automated analysis software is also included. (**E**) Quantification of ELISpot results including total IgM^+^ (left panel), *T*. *brucei*-specific IgM^+^ (middle panel), and mouse brain-specific IgM^+^ antibody secreting cells (right panel) in naïve mice, mice treated with an irrelevant IgG2a antibody, and mice treated with LTβR-Ig (*n* = 4–9 mice/group). A *p* value < 0.05 was considered significant. Supporting data in S36 Data file. **(F)** Luxol fast blue (LFB) staining to determine myelination in sagittal brain sections from infected mice treated with LTBR-Ig or with an irrelevant IgG2a antibody control naïve (2 replicates per condition). Insets how selected cortical areas. Ctx, Cortex. Scale bar: 1 mm (whole image) or 50 μm (insets).
(TIF)

S8 Fig Targeted antigen screening identified shared host antigens detected by autoreactive antibodies in mouse serum and human CSF in response to *T*. *brucei* infection. Heatmap depicting the normalised signal-to-noise ratio for IgG binding against a panel of 120 host antigens. The data was acquired from naïve and infected murine serum (**A**) at 30 dpi (*n* = 3 mice/group) and human CSF (B) from healthy donors (*n* = 2 donors), first stage sleeping sickness patients (*n* = 3 samples) and second stage sleeping sickness patients (*n* = 4 samples). The asterisks denote samples that were statistically significant in a pairwise comparison analysis using parametric *T* test. A *p* value < 0.05 is considered significant.
(EPS)

S1 Table Quality control including mean reads per cell and median genes per cell before and after filtering out low-quality cell types. (XLSX)

S2 Table Overview of the major cell types detected in the single-cell dataset (resolution of 0.7). The total number of cells per cluster, percentages, and marker genes are also included. (XLSX)

S3 Table Marker genes identified for the cells within the fibroblast clusters obtained after subsetting (resolution = 0.6). The total number of cells per cluster, percentages, and marker genes are also included. (XLSX)

S4 Table Marker genes identified for the cells within the mononuclear phagocytes (MNP) cluster obtained after subsetting (resolution = 0.3). The total number of cells per cluster, percentages, and marker genes are also included.
(XLSX)

S5 Table Marker genes identified for the cells within the T cell cluster obtained after subsetting (resolution = 0.7). The total number of cells per cluster, percentages, and marker genes are also included.
(XLSX)

S6 Table Metadata associated with the human cerebrospinal fluid samples included for autoantibodies against human brain lysates by ELISA. (XLSX)

S7 Table Normalised signal-to-noise ratio (SNR) of targeted host antigen screening conducted using mouse serum and human CSF. The SNR values for each of the 120 antigens are included, as well as a parametric *T* test conducted to determine the level of significant in pairwise comparisons between infected mice and naïve controls, or between second stage sleeping sickness CSF samples and first stage sleeping sickness or healthy donors.
(XLSX)

S1 Data Supporting data for Fig 1E—Quantification of CD45^+^ cells in the meninges by CyTOF. (XLSX)

S2 Data Supporting data for Fig 1F—Number of immune cell subsets as quantified by CyTOF. (XLSX)

S3 Data Supporting data for Fig 1G—Frequency of various immune cell subsets in the meninges quantified by CyTOF. (XLSX)

S4 Data Supporting data for Fig 2C—Frequency of meningeal cells derived from single-cell transcriptomics analysis. (XLSX)

S5 Data Supporting data for Fig 3D—Frequency of cells within the meningeal fibroblast subsets derived from single-cell transcriptomics analysis. (XLSX)

S6 Data Supporting data for Fig 3F—Quantification of meningeal stromal cells by flow cytometry. (XLSX)

S7 Data Supporting data for Fig 4C—Frequency of cells within the mononuclear phagocyte (MNPs) subset derived from single-cell transcriptomics analysis. (XLSX)

S8 Data Supporting data for Fig 4F—Frequency of various meningeal MNPs measured by flow cytometry. (XLSX)

S9 Data Supporting data for Fig 5E—Quantification of (percentage of) PD1^+^ CXCR5^+^ CD4^+^ T cells in the meninges from naïve and infected animals by flow cytometry. (XLSX)

S10 Data Supporting data for Fig 5G—Ex vivo stimulation assay to quantify (percentage of) PD1^+^ IL-21^+^ CD4^+^ T cells in the meninges from naïve and infected animals. (XLSX)

S11 Data Supporting data for Fig 5I—Quantification of CD69^+^ CD4^+^ T cells in the murine meninges during infection by flow cytometry. (XLSX)

S12 Data Supporting data for Fig 5K—Quantification of local T cell activation in the murine meninges using the Nur77^GFP^ reporter mice. (XLSX)

S13 Data Supporting data for Fig 5M—Quantification of local T cell activation in the murine meninges using the Nur77^Tempo^ reporter mice. (XLSX)

S14 Data Supporting data for Fig 6B—Quantification of meningeal CD138^+^ plasma cells (PCs) during infection by flow cytometry. (XLSX)

S15 Data Supporting data for Fig 6D—Quantification of local B cell activation and GC-like phenotype during infection by flow cytometry using the Nur77^GFP^ reporter mice. (XLSX)

S16 Data Supporting data for Fig 6F—Quantification of CD95^+^ GL7^+^ GC-like B cell phenotype during infection by flow cytometry. (XLSX)

S17 Data Supporting data for Fig 6H—Quantification of meningeal IgG^+^ CD19^+^ B cells during infection by flow cytometry. (XLSX)

S18 Data Supporting data for Fig 7B—Quantification of IgG antibody secreting cells (ASCs) by ELISpot against *T*. *brucei* lysate, mouse brain lysates, or BSA. The total IgG ASCs is also included. (XLSX)

S19 Data Supporting data for Fig 7D—Relative myelin intensity based on Luxol fast blue quantification from naïve and infected brain specimens. (XLSX)

S20 Data Supporting data for Fig 7E—Serum IgM and IgG ELISA test against mouse brain lysates from naïve and infected animals. (XLSX)

S21 Data Supporting data for Fig 7G—IgM and IgG ELISA test against human brain lysates from CSF samples obtained from first and second stage HAT patients. (XLSX)

S22 Data Supporting data for Fig 8C—Quantification of LTβ^+^ CD4^+^ T cells in the meninges from naïve and infected animals by flow cytometry. (XLSX)

S23 Data Supporting data for Fig 8F—Quantification of meningeal stromal cells by flow cytometry upon treatment with LTBR-Ig antibody. (XLSX)

S24 Data Supporting data for Fig 7B—Quantification of IgG antibody secreting cells (ASCs) by ELISpot against *T*. *brucei* lysate, mouse brain lysates upon treatment with LTBR-Ig. (XLSX)

S25 Data Supporting data for Fig 8I—Relative myelin intensity (%) based on Luxol fast blue quantification from naïve and infected brain specimens. (XLSX)

S26 Data Supporting data for Fig 9D—Human serum ELISA from HAT cases and treated patients to measure the levels of circulating antibodies against myelin basic protein (MBP). (XLSX)

S27 Data Supporting data for Fig 10B—Parasite quantification in the brain upon suramin treatment by qPCR to measure the *T*. *brucei*-specific transcript *Pfr2*. (XLSX)

S28 Data Supporting data for Fig 10E—Quantification of CD95^+^ GL7^+^ GC-like B cell phenotype upon suramin treatment by flow cytometry. (XLSX)

S29 Data Supporting data for Fig 10G—Mouse serum IgG against mouse brain lysates upon suramin treatment. (XLSX)

S30 Data Supporting data for Fig 10I—Relative myelin intensity (%) based on Luxol fast blue quantification from brain specimens upon suramin treatment. (XLSX)

S31 Data Supporting data for S4B Fig—Local CD8^+^ T cell activation in the meninges using the Nur77^Tempo^ reporter mice. (XLSX)

S32 Data Supporting data for S4D Fig—Local CD69^+^ CD4^+^ T cell activation in the meninges using the Nur77Tempo reporter mice. (XLSX)

S33 Data Supporting data for S6A Fig—Comparison of antibody secreting cells in the meninges and splenocytes against mouse brain lysate. (XLSX)

S34 Data Supporting data for S7A Fig—Parasitaemia of mice treated with LTBR-Ig blocking antibody of IgG2a isotype control (<5.4 falls below detection limit). (XLSX)

S35 Data Supporting data for S7B Fig—Clinical scoring of mice treated with LTBR-Ig blocking antibody of IgG2a isotype control. (XLSX)

S36 Data Supporting data for S7E Fig—Comparison of IgM antibody secreting cells (ASCs) in the meninges against *T*. *brucei* or mouse brain lysates. Total IgM ASCs are also included. (XLSX)

## Abbreviations

ASC: antibody secreting cell
BAM: border-associated macrophage
BEC: blood endothelial cell
CNS: central nervous system
CSF: cerebrospinal fluid
CyTOF: cytometry by time of flight
DC: dendritic cell
DEM: digital expression matrix
DMEM: Dubecco’s Modified Eagle Medium
dpi: days postinfection
EAE: experimental autoimmune encephalomyelitis
ECM: extracellular matrix
ELA: ectopic lymphoid aggregate
FDC: follicular dendritic cell
FRC: fibroblast reticular cell
FT: fluorescent timer
GC: germinal centre
HAT: human African trypanosomiasis
LEC: lymphatic endothelial cell
LFB: Luxol fast blue
maMNP: metabolically active mononuclear phagocyte
MBP: myelin basic protein
MDM: monocyte-derived macrophage
MNP: mononuclear phagocyte
PE: phycoerythrin
smFISH: single-molecule fluorescent in situ hybridisation
UMI: unique molecular identifier

## References

[1] Jacob L, de Brito Neto J, Lenck S, Corcy C, Benbelkacem F, Geraldo LH, et al. Conserved meningeal lymphatic drainage circuits in mice and humans. J Exp Med. 2022 Jul 1;219(8):e20220035. doi: 10.1084/jem.20220035 35776089 PMC9253621

[2] Rustenhoven J, Drieu A, Mamuladze T, de Lima KA, Dykstra T, Wall M, et al. Functional characterization of the dural sinuses as a neuroimmune interface. Cell. 2021 Feb 18;184(4):1000–1016.e27. doi: 10.1016/j.cell.2020.12.040 33508229 PMC8487654

[3] Antila S, Karaman S, Nurmi H, Airavaara M, Voutilainen MH, Mathivet T, et al. Development and plasticity of meningeal lymphatic vessels. J Exp Med. 2017 Nov 15;214(12):3645–67. doi: 10.1084/jem.20170391 29141865 PMC5716035

[4] Rustenhoven J, Pavlou G, Storck SE, Dykstra T, Du S, Wan Z, et al. Age-related alterations in meningeal immunity drive impaired CNS lymphatic drainage. J Exp Med. 2023 Apr 7;220(7):e20221929. doi: 10.1084/jem.20221929 37027179 PMC10083715

[5] Bolte AC, Shapiro DA, Dutta AB, Ma WF, Bruch KR, Kovacs MA, et al. The meningeal transcriptional response to traumatic brain injury and aging. Ginhoux F, Büchel C, Kim BS, editors. elife. 2023 Jan 3;12:e81154. doi: 10.7554/eLife.81154 36594818 PMC9810333

[6] Ma T, Wang F, Xu S, Huang JH. Meningeal immunity: Structure, function and a potential therapeutic target of neurodegenerative diseases. Brain Behav Immun. 2021 Mar 1;93:264–76. doi: 10.1016/j.bbi.2021.01.028 33548498

[7] Merlini A, Haberl M, Strauß J, Hildebrand L, Genc N, Franz J, et al. Distinct roles of the meningeal layers in CNS autoimmunity. Nat Neurosci. 2022 Jul;25(7):887–99. doi: 10.1038/s41593-022-01108-3 35773544

[8] Ampie L, McGavern DB. Immunological defense of CNS barriers against infections. Immunity. 2022 May 10;55(5):781–99. doi: 10.1016/j.immuni.2022.04.012 35545028 PMC9087878

[9] Coles JA, Myburgh E, Ritchie R, Hamilton A, Rodgers J, Mottram JC, et al. Intravital Imaging of a Massive Lymphocyte Response in the Cortical Dura of Mice after Peripheral Infection by Trypanosomes. PLoS Negl Trop Dis. 2015 Apr 16;9(4):e0003714. doi: 10.1371/journal.pntd.0003714 25881126 PMC4400075

[10] Vlaminck KD, Hove HV, Kancheva D, Scheyltjens I, Antunes ARP, Bastos J, et al. Differential plasticity and fate of brain-resident and recruited macrophages during the onset and resolution of neuroinflammation. Immunity. 2022 Nov 8;55(11):2085–2102.e9. doi: 10.1016/j.immuni.2022.09.005 36228615

[11] Coles JA, Stewart-Hutchinson PJ, Myburgh E, Brewer JM. The mouse cortical meninges are the site of immune responses to many different pathogens, and are accessible to intravital imaging. Methods San Diego Calif. 2017 Aug 15;127:53–61. doi: 10.1016/j.ymeth.2017.03.020 28351758 PMC5595162

[12] Rodgers J, Bradley B, Kennedy PGE. Delineating neuroinflammation, parasite CNS invasion, and blood-brain barrier dysfunction in an experimental murine model of human African trypanosomiasis. Methods San Diego Calif. 2017 Aug 15;127:79–87. doi: 10.1016/j.ymeth.2017.06.015 28636879 PMC5595161

[13] Quintana JF, Chandrasegaran P, Sinton MC, Briggs EM, Otto TD, Heslop R, et al. Single cell and spatial transcriptomic analyses reveal microglia-plasma cell crosstalk in the brain during Trypanosoma brucei infection. Nat Commun. 2022 Sep 30;13(1):5752. doi: 10.1038/s41467-022-33542-z 36180478 PMC9525673

[14] Blum J, Schmid C, Burri C. Clinical aspects of 2541 patients with second stage human African trypanosomiasis. Acta Trop. 2006 Jan;97(1):55–64. doi: 10.1016/j.actatropica.2005.08.001 16157286

[15] Kazumba LM, Kaka JCT, Ngoyi DM, Tshala-Katumbay D. Mortality trends and risk factors in advanced stage-2 Human African Trypanosomiasis: A critical appraisal of 23 years of experience in the Democratic Republic of Congo. PLoS Negl Trop Dis. 2018 Jun;12(6):e0006504. doi: 10.1371/journal.pntd.0006504 29897919 PMC5999091

[16] Mudji J, Blum A, Grize L, Wampfler R, Ruf MT, Cnops L, et al. Gambiense Human African Trypanosomiasis Sequelae after Treatment: A Follow-Up Study 12 Years after Treatment. Trop Med Infect Dis. 2020 Jan 11;5(1):10. doi: 10.3390/tropicalmed5010010 31940846 PMC7157708

[17] Rijo-Ferreira F, Bjorness TE, Cox KH, Sonneborn A, Greene RW, Takahashi JS. Sleeping Sickness Disrupts the Sleep-Regulating Adenosine System. J Neurosci. 2020 Nov 25;40(48):9306–16. doi: 10.1523/JNEUROSCI.1046-20.2020 33097636 PMC7687053

[18] Rijo-Ferreira F, Carvalho T, Afonso C, Sanches-Vaz M, Costa RM, Figueiredo LM, et al. Sleeping sickness is a circadian disorder. Nat Commun. 2018 Jan 4;9:62. doi: 10.1038/s41467-017-02484-2 29302035 PMC5754353

[19] Lundkvist GB, Hill RH, Kristensson K. Disruption of Circadian Rhythms in Synaptic Activity of the Suprachiasmatic Nuclei by African Trypanosomes and Cytokines. Neurobiol Dis. 2002 Oct 1;11(1):20–7. doi: 10.1006/nbdi.2002.0536 12460543

[20] Cornford EM, Freeman BJ, MacInnis AJ. Physiological Relationships and Circadian Periodicities in Rodent Trypanosomes. Trans R Soc Trop Med Hyg. 1976 Jan 1;70(3):238–43. doi: 10.1016/0035-9203(76)90047-x 982519

[21] Manzo A, Paoletti S, Carulli M, Blades MC, Barone F, Yanni G, et al. Systematic microanatomical analysis of CXCL13 and CCL21 in situ production and progressive lymphoid organization in rheumatoid synovitis. Eur J Immunol. 2005 May;35(5):1347–59. doi: 10.1002/eji.200425830 15832291

[22] Chang A, Henderson SG, Brandt D, Liu N, Guttikonda R, Hsieh C, et al. In situ B cell-mediated immune responses and tubulointerstitial inflammation in human lupus nephritis. J Immunol. 2011 Feb 1;186(3):1849–60. doi: 10.4049/jimmunol.1001983 21187439 PMC3124090

[23] Fridman WH, Meylan M, Petitprez F, Sun CM, Italiano A, Sautès-Fridman C. B cells and tertiary lymphoid structures as determinants of tumour immune contexture and clinical outcome. Nat Rev Clin Oncol. 2022 Jul;19(7):441–57. doi: 10.1038/s41571-022-00619-z 35365796

[24] Pitzalis C, Jones GW, Bombardieri M, Jones SA. Ectopic lymphoid-like structures in infection, cancer and autoimmunity. Nat Rev Immunol. 2014 Jul;14(7):447–62. doi: 10.1038/nri3700 24948366

[25] Stock AD, Der E, Gelb S, Huang M, Weidenheim K, Ben-Zvi A, et al. Tertiary lymphoid structures in the choroid plexus in neuropsychiatric lupus. JCI Insight. 2019;4(11):e124203. doi: 10.1172/jci.insight.124203 31167973 PMC6629135

[26] Pikor NB, Prat A, Bar-Or A, Gommerman JL. Meningeal Tertiary Lymphoid Tissues and Multiple Sclerosis: A Gathering Place for Diverse Types of Immune Cells during CNS Autoimmunity. Front Immunol. 2016. doi: 10.3389/fimmu.2015.00657 26793195 PMC4710700

[27] Rodriguez AB, Peske JD, Woods AN, Leick KM, Mauldin IS, Meneveau MO, et al. Immune mechanisms orchestrate tertiary lymphoid structures in tumors via cancer-associated fibroblasts. Cell Rep. 2021 Jul;36(3):109422. doi: 10.1016/j.celrep.2021.109422 34289373 PMC8362934

[28] Ransohoff RM. Multiple sclerosis: role of meningeal lymphoid aggregates in progression independent of relapse activity. Trends Immunol. 2023 Apr 1;44(4):266–75. doi: 10.1016/j.it.2023.02.002 36868982

[29] Jalkanen S, Salmi M. Lymphatic endothelial cells of the lymph node. Nat Rev Immunol. 2020 Sep;20(9):566–78. doi: 10.1038/s41577-020-0281-x 32094869

[30] Krishnamurty AT, Turley SJ. Lymph node stromal cells: cartographers of the immune system. Nat Immunol. 2020 Apr;21(4):369–80. doi: 10.1038/s41590-020-0635-3 32205888

[31] von Andrian UH, Mempel TR. Homing and cellular traffic in lymph nodes. Nat Rev Immunol. 2003 Nov;3(11):867–78. doi: 10.1038/nri1222 14668803

[32] Phan TG, Grigorova I, Okada T, Cyster JG. Subcapsular encounter and complement-dependent transport of immune complexes by lymph node B cells. Nat Immunol. 2007 Sep;8(9):992–1000. doi: 10.1038/ni1494 17660822

[33] Da Mesquita S, Fu Z, Kipnis J. The Meningeal Lymphatic System: A New Player in Neurophysiology. Neuron. 2018 Oct 24;100(2):375–88. doi: 10.1016/j.neuron.2018.09.022 30359603 PMC6268162

[34] Louveau A, Herz J, Alme MN, Salvador AF, Dong MQ, Viar KE, et al. CNS lymphatic drainage and neuroinflammation are regulated by meningeal lymphatic vasculature. Nat Neurosci. 2018 Oct;21(10):1380–91. doi: 10.1038/s41593-018-0227-9 30224810 PMC6214619

[35] Le Ray D, Barry JD, Easton C, Vickerman K. First tsetse fly transmission of the ‘AnTat’ serodeme of Trypanosoma brucei. Ann Soc Belg Med Trop. 1977;57(4–5):369–381. 610616

[36] Herbert WJ, Lumsden WH. Trypanosoma brucei: a rapid ‘matching’ method for estimating the host’s parasitemia. Exp Parasitol. 1976 Dec;40(3):427–31. doi: 10.1016/0014-4894(76)90110-7 976425

[37] Anderson K, Mayer-Barber K, Sung H. Intravascular staining for discrimination of vascular and tissue leukocytes. Nat Protoc. 2014;9:209–222. doi: 10.1038/nprot.2014.005 24385150 PMC4428344

[38] Hao Y, Hao S, Andersen-Nissen E, Mauck WM, Zheng S, Butler A, et al. Integrated analysis of multimodal single-cell data. Cell. 2021 Jun 24;184(13):3573–3587.e29. doi: 10.1016/j.cell.2021.04.048 34062119 PMC8238499

[39] Hafemeister C, Satija R. Normalization and variance stabilization of single-cell RNA-seq data using regularized negative binomial regression. Genome Biol. 2019 Dec 23;20(1):296. doi: 10.1186/s13059-019-1874-1 31870423 PMC6927181

[40] Zappia L, Oshlack A. Clustering trees: a visualization for evaluating clusterings at multiple resolutions. GigaScience. 2018 July;7(7):giy083. doi: 10.1093/gigascience/giy083 30010766 PMC6057528

[41] Efremova M, Vento-Tormo M, Teichmann SA, Vento-Tormo R. CellPhoneDB: inferring cell-cell communication from combined expression of multi-subunit ligand-receptor complexes. Nat Protoc. 2020 Apr;15(4):1484–506. doi: 10.1038/s41596-020-0292-x 32103204

[42] Brioschi S, Wang WL, Peng V, Wang M, Shchukina I, Greenberg ZJ, et al. Heterogeneity of meningeal B cells reveals a lymphopoietic niche at the CNS borders. Science. 2021 Jul 23;373(6553):eabf9277. doi: 10.1126/science.abf9277 34083450 PMC8448524

[43] Shao X, Taha IN, Clauser KR, Gao Y, Naba A. MatrisomeDB: the ECM-protein knowledge database. Nucleic Acids Res. 2020 Jan 8;48(D1):D1136–44. doi: 10.1093/nar/gkz849 31586405 PMC6943062

[44] Manglani M, Gossa S, McGavern DB. Leukocyte Isolation from Brain, Spinal Cord, and Meninges for Flow Cytometric Analysis. Curr Protoc Immunol. 2018 Apr;121(1):e44. doi: 10.1002/cpim.44 30040211 PMC6060638

[45] Browning JL, Sizing ID, Lawton P, Bourdon PR, Rennert PD, Majeau GR, et al. Characterization of lymphotoxin-alpha beta complexes on the surface of mouse lymphocytes. J Immunol Baltim Md. 1997 Oct 1;159(7):3288–98. 9317127

[46] Fitzpatrick Z, Frazer G, Ferro A, Clare S, Bouladoux N, Ferdinand J, et al. Gut-educated IgA plasma cells defend the meningeal venous sinuses. Nature. 2020;587(7834):472–476. doi: 10.1038/s41586-020-2886-4 33149302 PMC7748383

[47] Dorrier CE, Jones HE, Pintarić L, Siegenthaler JA, Daneman R. Emerging roles for CNS fibroblasts in health, injury and disease. Nat Rev Neurosci. 2022 Jan;23(1):23–34. doi: 10.1038/s41583-021-00525-w 34671105 PMC8527980

[48] DeSisto J, O’Rourke R, Jones HE, Pawlikowski B, Malek AD, Bonney S, et al. Single-Cell Transcriptomic Analyses of the Developing Meninges Reveal Meningeal Fibroblast Diversity and Function. Dev Cell. 2020 Jul;54(1):43–59.e4. doi: 10.1016/j.devcel.2020.06.009 32634398 PMC7769050

[49] Jones HE, Coelho-Santos V, Bonney SK, Abrams KA, Shih AY, Siegenthaler JA. Meningeal origins and dynamics of perivascular fibroblast development on the mouse cerebral vasculature. bioRxiv. 2023. https://www.biorxiv.org/content/10.1101/2023.03.23.533982v1 10.1242/dev.201805 PMC10565218 37756588

[50] Di Carlo SE, Peduto L. The perivascular origin of pathological fibroblasts. J Clin Invest. 2018;128(1):54–63. doi: 10.1172/JCI93558 29293094 PMC5749494

[51] Friede RL, Schachenmayr W. The origin of subdural neomembranes. I. Fine structure of the dura-arachnoid interface in man. Am J Pathol. 1978 Jul; 92(1):69–84. 686148 PMC2018597

[52] Schiavinato A, Przyklenk M, Kobbe B, Paulsson M, Wagener R. Collagen type VI is the antigen recognized by the ER-TR7 antibody. Eur J Immunol. 2021 Sep;51(9):2345–7. doi: 10.1002/eji.202149263 34180542

[53] Yoshihara T, Okabe Y. Aldh1a2+ fibroblastic reticular cells regulate lymphocyte recruitment in omental milky spots. J Exp Med. 2023;220(5):e20221813. doi: 10.1084/jem.20221813 36880532 PMC9997506

[54] DeSisto J, O’Rourke R, Bonney S, Jones HE, Guimiot F, Jones KL, et al. A cellular atlas of the developing meninges reveals meningeal fibroblast diversity and function. bioRxiv. 2019 Sept. https://www.biorxiv.org/content/10.1101/648642v1 10.1016/j.devcel.2020.06.009 PMC7769050 32634398

[55] Remsik J, Saadeh F, Tong X, Li MJ, Snyder J, Bale T, et al. Characterization, isolation, and in vitro culture of leptomeningeal fibroblasts. J Neuroimmunol. 2021 Dec; 15:361–577727. doi: 10.1016/j.jneuroim.2021.577727 34688068 PMC8648257

[56] Pietilä R, Del Gaudio F, He L, Vázquez-Liébanas E, Vanlandewijck M, Muhl L, et al. Molecular anatomy of adult mouse leptomeninges. Neuron. 2023;111:1–20. 37776854 10.1016/j.neuron.2023.09.002

[57] Cinti I, Denton AE. Lymphoid stromal cells—more than just a highway to humoral immunity. Oxf Open Immunol. 2021 Jan 1;2(1):iqab011. doi: 10.1093/oxfimm/iqab011 36845565 PMC9914513

[58] Lutge M, De Martin A, Gil-Cruz C, Perez-Shibayama C, Stanossek Y, Onder L, et al. Conserved stromal–immune cell circuits secure B cell homeostasis and function. Nat Immunol. 2023;24:1149–1160. doi: 10.1038/s41590-023-01503-3 37202489 PMC10307622

[59] Krautler NJ, Kana V, Kranich J, Tian Y, Perera D, Lemm D, et al. Follicular Dendritic Cells Emerge from Ubiquitous Perivascular Precursors. Cell. 2012 Jul 6;150(1):194–206. doi: 10.1016/j.cell.2012.05.032 22770220 PMC3704230

[60] McCulloch L, Brown KL, Bradford BM, Hopkins J, Bailey M, Rajewsky K, et al. Follicular Dendritic Cell-Specific Prion Protein (PrPc) Expression Alone Is Sufficient to Sustain Prion Infection in the Spleen. PLoS Pathog. 2011 Dec 1;7(12):e1002402. 22144895 10.1371/journal.ppat.1002402 PMC3228802

[61] Waddell LA, Lefevre L, Bush SJ, Raper A, Young R, Lisowski ZM, et al. ADGRE1 (EMR1, F4/80) Is a Rapidly-Evolving Gene Expressed in Mammalian Monocyte-Macrophages. Front Immunol. 2018;9:2246. doi: 10.3389/fimmu.2018.02246 30327653 PMC6174849

[62] Van Hove H, Martens L, Scheyltjens I, De Vlaminck K, Pombo Antunes AR, De Prijck S, et al. A single-cell atlas of mouse brain macrophages reveals unique transcriptional identities shaped by ontogeny and tissue environment. Nat Neurosci. 2019 Jun;22(6):1021–35. doi: 10.1038/s41593-019-0393-4 31061494

[63] Moran AE, Holzapfel KL, Xing Y, Cunningham NR, Maltzman JS, Punt J, et al. T cell receptor signal strength in Treg and iNKT cell development demonstrated by a novel fluorescent reporter mouse. J Exp Med. 2011 Jun 6;208(6):1279–89. doi: 10.1084/jem.20110308 21606508 PMC3173240

[64] Elliot TAE, Jennings EK, Lecky DAJ, Rouvray S, Mackie GM, Scarfe L, et al. Nur77-Tempo mice reveal T cell steady state antigen recognition. Discov Immunol. 2022 Jan 1;1(1):kyac009. doi: 10.1093/discim/kyac009 36704407 PMC7614040

[65] Jennings E, Elliot TAE, Thawait N, Kanabar S, Yam-Puc JC, Ono M, et al. Nr4a1 and Nr4a3 Reporter Mice Are Differentially Sensitive to T Cell Receptor Signal Strength and Duration. Cell Rep. 2020;33(5):108328. doi: 10.1016/j.celrep.2020.108328 33147449 PMC7653457

[66] Barinov A, Luo L, Gasse P, Meas-Yedid V, Donnadieu E, Arenzana-Seisdedos F, et al. Essential role of immobilized chemokine CXCL12 in the regulation of the humoral immune response. Proc Natl Acad Sci U S A. 2017 Feb 28;114(9):2319–24. doi: 10.1073/pnas.1611958114 28193885 PMC5338526

[67] Cheng Q, Khodadadi L, Taddeo A, Klotsche J. CXCR4–CXCL12 interaction is important for plasma cell homing and survival in NZB/W mice. Eur J Immunol. 2018;48(6):1020–1029. doi: 10.1002/eji.201747023 29427452

[68] Pilzecker B, Jacobs H. Mutating for Good: DNA Damage Responses During Somatic Hypermutation. Front Immunol. 2019 March;10. doi: 10.3389/fimmu.2019.00438 30915081 PMC6423074

[69] Mueller J, Matloubian M, Zikherman J. An in vivo reporter reveals active B cell receptor signaling in the germinal center. J Immunol. 2015 Apr 1;194(7):2993–7. 25725108 10.4049/jimmunol.1403086 PMC4369439

[70] Brooks JF, Tan C, Mueller JL, Hibiya K, Hiwa R, Vykunta V, et al. Negative feedback by NUR77/Nr4a1 restrains B cell clonal dominance during early T-dependent immune responses. Cell Rep. 2021 Aug 31;36(9):109645. doi: 10.1016/j.celrep.2021.109645 34469720 PMC8564879

[71] Kumar Dubey L, Lebon L, Mosconi I, Yang CY, Scandella E, Ludewig B, et al. Lymphotoxin-Dependent B Cell-FRC Crosstalk Promotes De Novo Follicle Formation and Antibody Production following Intestinal Helminth Infection. Cell Rep. 2016 May; 15(7):1527–1541. doi: 10.1016/j.celrep.2016.04.023 27160906

[72] Tang H, Zhu M, Qiao J, Fu YX. Lymphotoxin signalling in tertiary lymphoid structures and immunotherapy. Cell Mol Immunol. 2017 Oct;14(10):809–18. doi: 10.1038/cmi.2017.13 28413217 PMC5649108

[73] Koroleva EP, Fu YX, Tumanov AV. Lymphotoxin in physiology of lymphoid tissues—implication for antiviral defense. Cytokine. 2018 Jan;101:39–47. doi: 10.1016/j.cyto.2016.08.018 27623349 PMC5344785

[74] James Bates RE, Browne E, Schalks R, Jacobs H, Tan L, Parekh P, et al. Lymphotoxin-alpha expression in the meninges causes lymphoid tissue formation and neurodegeneration. Brain. 2022 Dec 1;145(12):4287–307. doi: 10.1093/brain/awac232 35776111 PMC9762953

[75] Browning JL, Allaire N, Ngam-ek A, Notidis E, Hunt J, Perrin S, et al. Lymphotoxin-β Receptor Signaling Is Required for the Homeostatic Control of HEV Differentiation and Function. Immunity. 2005 Nov 1;23(5):539–50. 16286021 10.1016/j.immuni.2005.10.002

[76] Alfituri OA, Bradford BM, Paxton E, Morrison LJ, Mabbott NA. Influence of the Draining Lymph Nodes and Organized Lymphoid Tissue Microarchitecture on Susceptibility to Intradermal Trypanosoma brucei Infection. Front Immunol. 2020;11:1118. doi: 10.3389/fimmu.2020.01118 32582198 PMC7283954

[77] Pisetsky DS. Pathogenesis of autoimmune disease. Nat Rev Nephrol. 2023 Aug;19(8):509–24. doi: 10.1038/s41581-023-00720-1 37165096 PMC10171171

[78] Zhang F, Gao X, Liu J, Zhang C. Biomarkers in autoimmune diseases of the central nervous system. Front Immunol. 2023 Sept; 14:doi: 10.3389/fimmu.2023.1111719 37090723 PMC10113662

[79] Theofilopoulos AN, Kono DH, Baccala R. The multiple pathways to autoimmunity. Nat Immunol. 2017 Jul;18(7):716–24. doi: 10.1038/ni.3731 28632714 PMC5791156

[80] Amin DN, Masocha W, Ngan’dwe K, Rottenberg M, Kristensson K. Suramin and minocycline treatment of experimental African trypanososmiasis at an early stage of parasite brain invasion. Acta Trop. 2008 Apr 1;106(1):72–4. doi: 10.1016/j.actatropica.2008.01.005 18329619

[81] Buechler MB, Pradhan RN, Krishnamurty AT, Cox C, Calviello AK, Wang AW, et al. Cross-tissue organization of the fibroblast lineage. Nature. 2021 May;593(7860):575–9. doi: 10.1038/s41586-021-03549-5 33981032

[82] Lendahl U, Muhl L, Betsholtz C. Identification, discrimination and heterogeneity of fibroblasts. Nat Commun. 2022 Jun 14;13:3409. doi: 10.1038/s41467-022-30633-9 35701396 PMC9192344

[83] Plikus MV, Wang X, Sinha S, Forte E, Thompson SM, Herzog EL, et al. Fibroblasts: Origins, definitions, and functions in health and disease. Cell. 2021 Jul;184(15):3852–72. doi: 10.1016/j.cell.2021.06.024 34297930 PMC8566693

[84] Denton A, Dooley J, Cinti I, Silva-Cayetano A, Fra-Bido S, Innocentin S, et al. Targeting TLR4 during vaccination boosts MAdCAM-1+ lymphoid stromal cell activation and promotes the aged germinal center response. Science Immunol. 2022 May; 7(71):doi: 10.1126/sciimmunol.abk0018 35522725 PMC7612953

[85] Hsu M, Rayasam A, Kijak JA, Choi YH, Harding JS, Marcus SA, et al. Neuroinflammation-induced lymphangiogenesis near the cribriform plate contributes to drainage of CNS-derived antigens and immune cells. Nat Commun. 2019 Jan 16;10(1):229. doi: 10.1038/s41467-018-08163-0 30651548 PMC6335416

[86] Li Z, Antila S, Nurmi H, Chilov D, Korhonen EA, Fang S. Blockade of VEGFR3 signaling leads to functional impairment of dural lymphatic vessels without affecting autoimmune neuroinflammation | Science Immunol. 2023 Apr; 8(82):eabq0375. 10.1126/sciimmunol.abq0375 37058549

[87] Bjornevik K, Cortese M, Healy BC, Khule J, Leng Y, Elledge SJ, et al. Longitudinal analysis reveals high prevalence of Epstein-Barr virus associated with multiple sclerosis. Science. 2023 Apr; 375(6578). doi: 10.1126/science.abj8222 35025605

[88] Guillaume MP, Hermanus N, Demulder A, Servais G, Karmali R. Specific autoantibodies of SLE, such as anti-Ku, anti-ribosome Po and anti-membrane DNA autoantibodies, in a case of human African trypanosomiasis. Rheumatology. 2003 Dec 1;42(12):1568–9. doi: 10.1093/rheumatology/keg390 14645862

[89] Radwanska M, Magez S, Dumont N, Pays A, Nolan D, Pays E. Antibodies raised against the flagellar pocket fraction of Trypanosoma brucei preferentially recognize HSP60 in cDNA expression library. Parasite Immunol. 2000;22(12):639–650. doi: 10.1046/j.1365-3024.2000.00348.x 11123756

[90] Radwanska M, Magez S, Michel A, Stijlemans B, Geuskens M, Pays E. Comparative Analysis of Antibody Responses against HSP60, Invariant Surface Glycoprotein 70, and Variant Surface Glycoprotein Reveals a Complex Antigen-Specific Pattern of Immunoglobulin Isotype Switching during Infection by Trypanosoma brucei. Mansfield JM, editor. Infect Immun. 2000 Feb;68(2):848–860. doi: 10.1128/IAI.68.2.848-860.2000 10639455 PMC97214

[91] Ayed Z, Brindel I, Bouteille B, Meirvenne NV, Doua F, Houinato D, et al. Detection and Characterization of Autoantibodies Directed against Neurofilament Proteins in Human African Trypanosomiasis. Am J Trop Med Hyg. 1997 Jul 1;57(1):1–6. 9242309

[92] Kazyumba G, Berney M, Brighouse G, Cruchaud A, Lambert PH. Expression of the B cell repertoire and autoantibodies in human African trypanosomiasis. Clin Exp Immunol. 1986 Jul;65(1):10–8. 3491699 PMC1542291

[93] Schafflick D, Wolbert J, Heming M, Thomas C, Hartlehnert M, Börsch AL, et al. Single-cell profiling of CNS border compartment leukocytes reveals that B cells and their progenitors reside in non-diseased meninges. Nat Neurosci. 2021 Sep;24(9):1225–34. doi: 10.1038/s41593-021-00880-y 34253922

[94] Kovacs MA, Cowan MN, Babcock IW, Sibley LA, Still K, Batista SJ, et al. Meningeal lymphatic drainage promotes T cell responses against Toxoplasma gondii but is dispensable for parasite control in the brain. Ginhoux F, Diamond B, Mundt S, editors. elife. 2022 Dec 21;11:e80775. doi: 10.7554/eLife.80775 36541708 PMC9812409

[95] De Niz M, Brás D, Ouarné M, Pedro M, Nascimento AM, Henao Misikova L, et al. Organotypic endothelial adhesion molecules are key for Trypanosoma brucei tropism and virulence. Cell Rep. 2021 Sep;36(12):109741. doi: 10.1016/j.celrep.2021.109741 34551286 PMC8480282

[96] Philip KA, Dascombe MJ, Fraser PA, Pentreath VW. Blood-brain barrier damage in experimental African trypanosomiasis. Ann Trop Med Parasitol. 1994 Dec;88(6):607–16. doi: 10.1080/00034983.1994.11812911 7893174

[97] Rodgers J, McCabe C, Gettinby G, Bradley B, Condon B, Kennedy PGE. Magnetic resonance imaging to assess blood-brain barrier damage in murine trypanosomiasis. Am J Trop Med Hyg. 2011 Feb;84(2):344–50. doi: 10.4269/ajtmh.2011.10-0487 21292912 PMC3029195

[98] Fillatreau S, Manfroi B, Dörner T. Toll-like receptor signalling in B cells during systemic lupus erythematosus. Nat Rev Rheumatol. 2021 Feb;17(2):98–108. doi: 10.1038/s41584-020-00544-4 33339987 PMC7747191

[99] Hua Z, Hou B. TLR signaling in B-cell development and activation. Cell Mol Immunol. 2013 Mar;10(2):103–6. doi: 10.1038/cmi.2012.61 23241902 PMC4003046

[100] Hartlehnert M, Börsch AL, Li X, Burmeister M, Gerwien H, Schafflick D, et al. Bcl6 controls meningeal Th17–B cell interaction in murine neuroinflammation. Proc Natl Acad Sci U S A. 2021 Sep 7;118(36):e2023174118. doi: 10.1073/pnas.2023174118 34479995 PMC8433502

[101] Baxter AG. The origin and application of experimental autoimmune encephalomyelitis. Nat Rev Immunol. 2007 Nov;7(11):904–12. doi: 10.1038/nri2190 17917672

[102] Fletcher JM, Lalor SJ, Sweeney CM, Tubridy N, Mills KHG. T cells in multiple sclerosis and experimental autoimmune encephalomyelitis. Clin Exp Immunol. 2010 Oct 1;162(1):1–11. doi: 10.1111/j.1365-2249.2010.04143.x 20682002 PMC2990924

[103] Lopez JA, Denkova M, Ramanathan S, Dale RC, Brilot F. Pathogenesis of autoimmune demyelination: from multiple sclerosis to neuromyelitis optica spectrum disorders and myelin oligodendrocyte glycoprotein antibody-associated disease. Clin Transl Immunol. 2021;10(7):e1316. doi: 10.1002/cti2.1316 34336206 PMC8312887

[104] Lucchinetti CF, Popescu BFG, Bunyan RF, Moll NM, Roemer SF, Lassmann H, et al. Inflammatory Cortical Demyelination in Early Multiple Sclerosis. N Engl J Med. 2011 Dec 8;365(23):2188–97. doi: 10.1056/NEJMoa1100648 22150037 PMC3282172

[105] Cikes N, Bosnic D, Sentic M. Non-MS autoimmune demyelination. Clin Neurol Neurosurg. 2008;110(9):905–912. doi: 10.1016/j.clineuro.2008.06.011 18928881

[106] Nguyen HTT, Guevarra RB, Magez S, Radwanska M. Single-cell transcriptome profiling and the use of AID deficient mice reveal that B cell activation combined with antibody class switch recombination and somatic hypermutation do not benefit the control of experimental trypanosomosis. PLoS Pathog. 2021 Nov 11;17(11):e1010026. doi: 10.1371/journal.ppat.1010026 34762705 PMC8610246

[107] Friman V, Quinti I, Davydov AN, Shugay M, Farroni C, Engström E, et al. Defective peripheral B cell selection in common variable immune deficiency patients with autoimmune manifestations. Cell Rep. 2023 May;42(5). doi: 10.1016/j.celrep.2023.112446 37119135

[108] Magliozzi R, Marastoni D, Calabrese M. The BAFF / APRIL system as therapeutic target in multiple sclerosis. Expert Opin Ther Targets. 2020 Nov;24(11):1135–45. doi: 10.1080/14728222.2020.1821647 32900236

[109] Lee DSW, Rojas OL, Gommerman JL. B cell depletion therapies in autoimmune disease: advances and mechanistic insights. Nat Rev Drug Discov. 2021 Mar;20(3):179–99. doi: 10.1038/s41573-020-00092-2 33324003 PMC7737718

[110] Florou D, Katsara M, Feehan J, Dardiotis E, Apostolopoulos V. Anti-CD20 Agents for Multiple Sclerosis: Spotlight on Ocrelizumab and Ofatumumab. Brain Sci. 2020 Oct 20;10(10):758. doi: 10.3390/brainsci10100758 33092190 PMC7589300

[111] Blattner MS, de Bruin GS, Bucelli RC, Day GS. Sleep disturbances are common in patients with autoimmune encephalitis. J Neurol. 2019 Apr 1;266(4):1007–15. doi: 10.1007/s00415-019-09230-2 30741377 PMC6421090

[112] Blattner MS, Day GS. Sleep Disturbances in Patients with Autoimmune Encephalitis. Curr Neurol Neurosci Rep. 2020 Jun 10;20(7):28. doi: 10.1007/s11910-020-01048-0 32524504 PMC7295028

[113] Yin D, Chen S, Liu J. Sleep Disturbances in Autoimmune Neurologic Diseases: Manifestation and Pathophysiology. Front Neurosci. 2021 Aug 6;15:687536. doi: 10.3389/fnins.2021.687536 34421519 PMC8377735

[114] Bonvalet M, Ollila HM, Ambati A, Mignot E. Autoimmunity in narcolepsy. Curr Opin Pulm Med. 2017;23(6):522–529. doi: 10.1097/MCP.0000000000000426 28991006 PMC5773260

[115] Mignot E, Lin L, Rogers W, Honda Y, Qiu X, Lin X, et al. Complex HLA-DR and -DQ Interactions Confer Risk of Narcolepsy-Cataplexy in Three Ethnic Groups. Am J Hum Genet. 2001 Mar 1;68(3):686–99. doi: 10.1086/318799 11179016 PMC1274481

