# Supplementary figures and images for: The murine meninges acquire lymphoid tissue properties and harbour autoreactive B cells during chronic Trypanosoma brucei infection
Source: PLoS Biol. 2023 Nov 20;21(11):e3002389. doi: 10.1371/journal.pbio.3002389 (PMC10723712; doi:10.1371/journal.pbio.3002389)

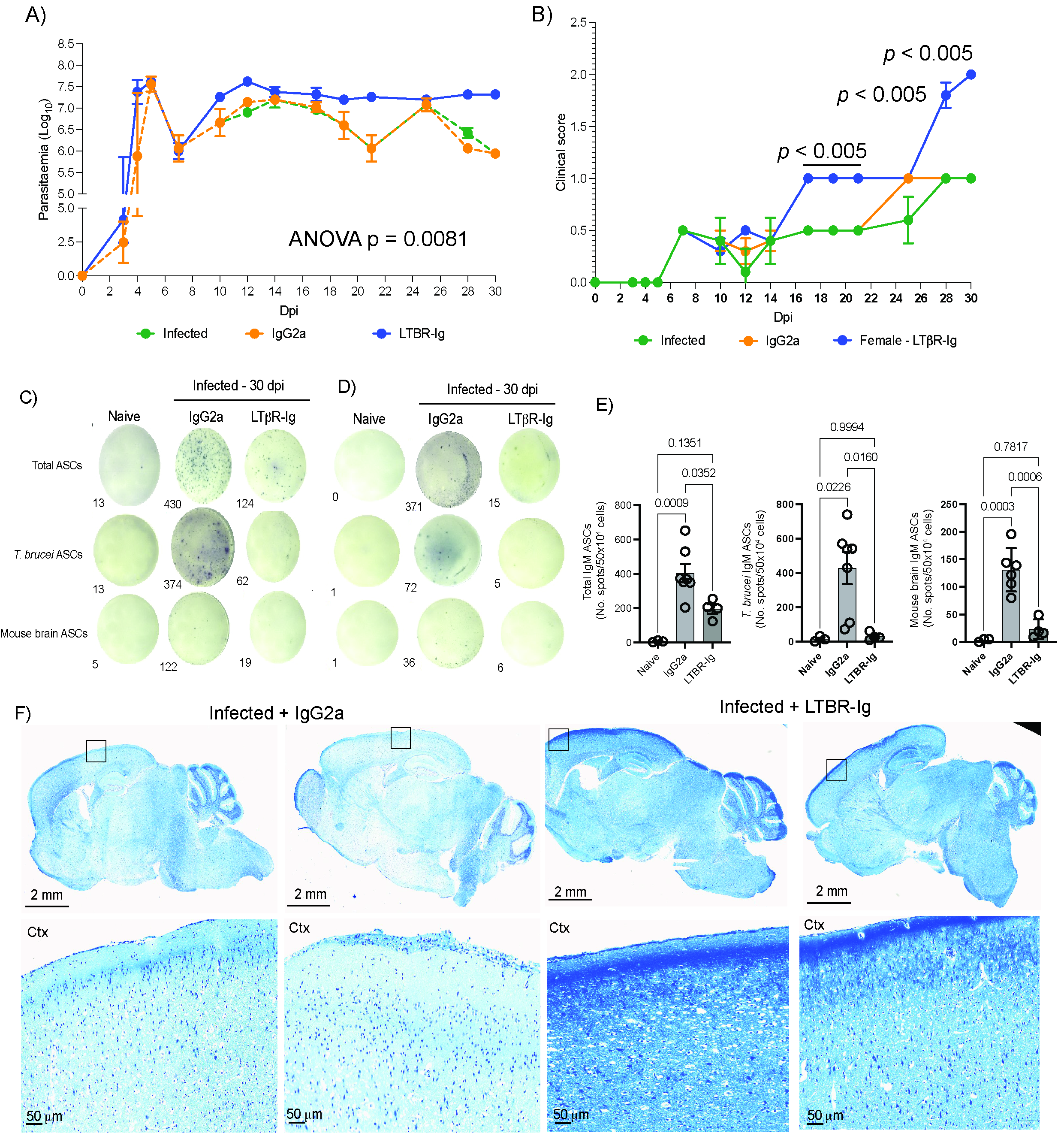

Supplement: S7 Fig — Parasitaemia (A) and clinical scoring (B) of T. brucei-infected mice treated with the LTβR-Ig fusion protein (blue line). T. brucei-infected mice alone (green line) or infected mice treated with an irrelevant IgG2a antibody (orange line) were used as controls. For parasitaemia, an ANOVA test with multiple comparisons was conducted. For clinical scoring, pairwise comparisons were conducted using a nonparametric T test. In all cases, a p value < 0.05 was considered significant. Supporting data in S34 and S35 Data file for parasitaemia and clinical scoring, respectively. Representative ELISpot results for meningeal IgM+ (C) and IgG+ (D) antibody secreting cells (ASCs), including total ASCs (top panel), T. brucei-specific ASCs (middle panel), and mouse brain-specific ASCs (bottom panel). The number of spots detected by the automated analysis software is also included. (E) Quantification of ELISpot results including total IgM+ (left panel), T. brucei-specific IgM+ (middle panel), and mouse brain-specific IgM+ antibody secreting cells (right panel) in naïve mice, mice treated with an irrelevant IgG2a antibody, and mice treated with LTβR-Ig (n = 4–9 mice/group). A p value < 0.05 was considered significant. Supporting data in S36 Data file. (F) Luxol fast blue (LFB) staining to determine myelination in sagittal brain sections from infected mice treated with LTBR-Ig or with an irrelevant IgG2a antibody control naïve (2 replicates per condition). Insets how selected cortical areas. Ctx, Cortex. Scale bar: 1 mm (whole image) or 50 μm (insets). (TIF) [file pbio.3002389.s007.tif]
